# 24.0 kyr cal BP stone artefact from Vale da Pedra Furada, Piauí, Brazil: Techno-functional analysis

**DOI:** 10.1371/journal.pone.0247965

**Published:** 2021-03-10

**Authors:** Eric Boëda, Marcos Ramos, Antonio Pérez, Christine Hatté, Christelle Lahaye, Mario Pino, David Hérisson, Ignacio Clemente-Conte, Michel Fontugne, Guillaume Guérin, Ximena Villagran, Janaina C. Santos, Lucas Costa, Lucie Germond, Nelson Eric Ahmed-Delacroix, Amelie Da Costa, Carolina Borges, Sirley Hoeltz, Gisele Felice, María Gluchy, Grégoire van Havre, Christophe Griggo, Livia Lucas, Iderlan de Souza, Sibeli Viana, André Strauss, Jennifer Kerner, Niède Guidon

**Affiliations:** 1 ArScAn-Équipe AnTET, UMR 7041, CNRS, Université Paris Nanterre (UPN), Nanterre, France; 2 Department of Anthropology, UFR SSA, Université Paris Nanterre (UPN), Nanterre, France; 3 PPGArq-Museu Nacional, Universidade Federal do Rio de Janeiro (UFRJ), Rio de Janeiro, Brazil; 4 Institut français d’études andines (IFEA), Lima, Peru; 5 LSCE/LAMPEA, UMR 8212, CNRS, CEA UVSQ, Université Paris-Saclay, Gif-sur-Yvette, France; 6 IRAMAT-CRP2A, UMR 5060, CNRS, Bordeaux Montaigne University, Pessac, France; 7 Instituto de Ciencias de la Tierra and TAQUACH, Universidad Austral de Chile, Valdivia, Chile; 8 Archaeology of Social Dynamics (2017SGR995), CSIC-IMF, Barcelona, Spain; 9 MAE–Museu de Arqueologia e Etnologia, Universidade de São Paulo (USP), São Paulo, Brazil; 10 Universidade Federal do Vale do São Francisco (UNIVASF), Petrolina, Brazil; 11 Universidade Federal do Piauí (UFPI), Teresina, Brazil; 12 Instituto do Patrimonio Histórico e Artístico Nacional (IPHAN), Piauí, Brazil; 13 Archaeo: Pesquisas Arqueológicas, Cuiabá/MT, Brazil; 14 Fundação Museu do Homem Americano (FUMDHAM), São Raimundo Nonato, Piauí, Brazil; 15 Universidade Federal do Rio Grande (FURG), Rio Grande, Brazil; 16 EDYTEM UMR 5204 CNRS, Université Savoie Mont Blanc, Le Bourget-du-Lac, France; 17 Universidade Federal de Sergipe (UFS), Sergipe, Brazil; 18 Pontificia Universidade Católica de Goiás (PUC-GO), Instituto Goiano de Pré-História e Antropologia (IGPA), Goiânia, Brazil; Universita degli Studi di Ferrara, ITALY

## Abstract

Current archaeological paradigm proposes that the first peopling of the Americas does not exceed the Last Glacial Maximum period. In this context, the acceptance of the anthropogenic character of the earliest stone artefacts generally rests on the presence of projectile points considered no more as typocentric but as typognomonic, since it allows, by itself, to certify the human character of the other associated artefacts. In other words, without this presence, nothing is certain. Archaeological research at Piauí (Brazil) attests to a Pleistocene human presence between 41 and 14 cal kyr BP, without any record of lithic projectile points. Here, we report the discovery and interpretation of an unusual stone artefact in the Vale da Pedra Furada site, in a context dating back to 24 cal kyr BP. The knapping stigmata and macroscopic use-wear traces reveal a conception centred on the configuration of double bevels and the production in the same specimen of at least two successive artefacts with probably different functions. This piece unambiguously presents an anthropic character and reveals a technical novelty during the Pleistocene occupation of South America.

## Introduction

The peopling of the Americas continues to be subject of intense debate, basically between two positions that often do not consider fully South American research: a Last Glacial Maximum (LGM *sensu stricto*, dated between 19–23 kyr BP [1]) occupation (current consensus) and a pre-LGM occupation. In support of the latter, a growing body of evidence demonstrates a Late Pleistocene human presence (i.e. Paleoamerican) in South America well beyond 20 cal kyr BP [2–28]. The timing of entry and settlement, the number of migratory waves, subsistence systems and the nature of early technologies also remain in dispute [27]. Within this context, the description and interpretation of lithic technology in South America is impregnated by the epistemological weight of the so-called projectile points [29], an inheritance of the already collapsed Clovis-First paradigm.

**Fig 1:**
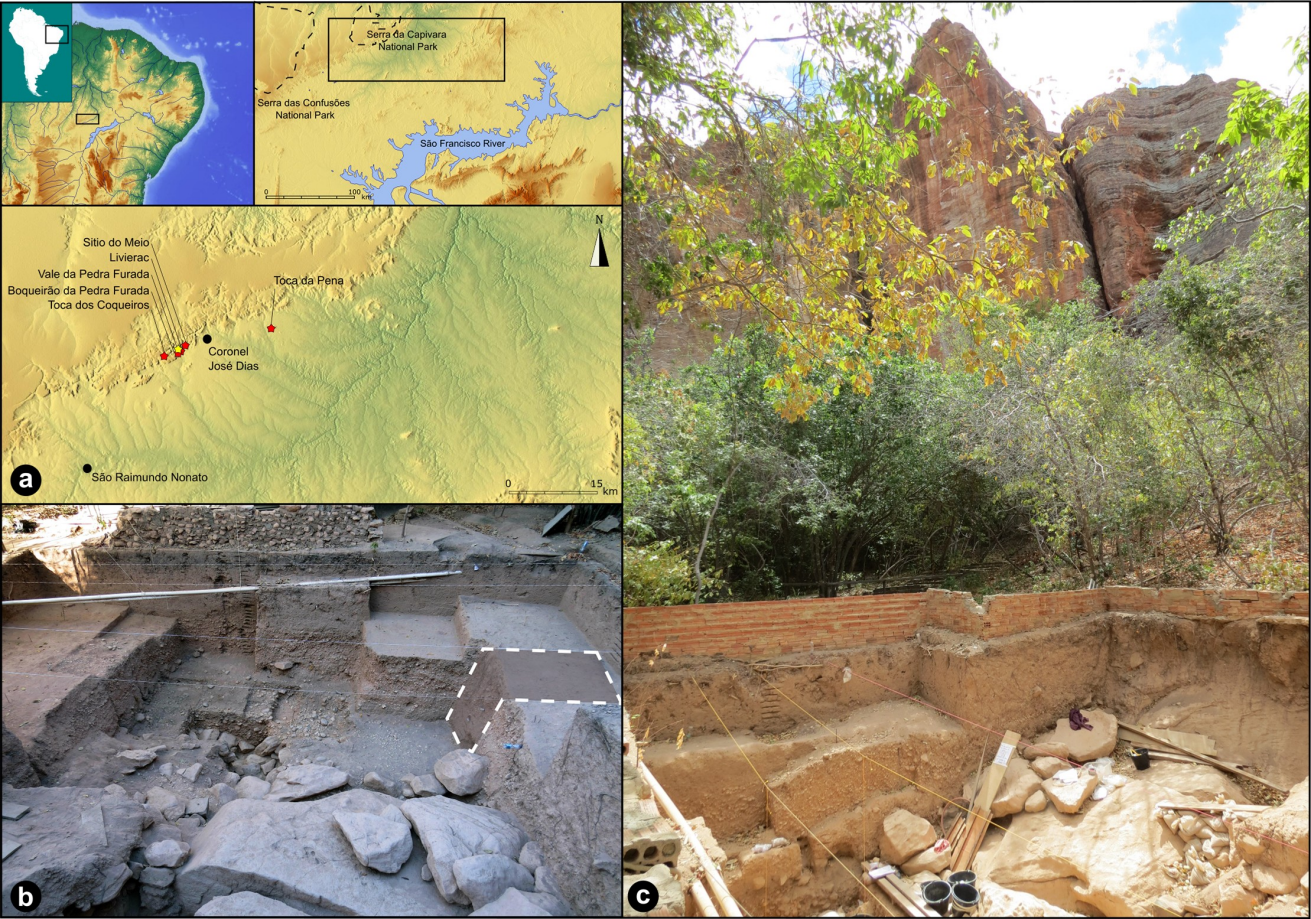
a. Location of the Vale da Pedra Furada (VPF) site. Map showing the spatial relation between VPF and other Pleistocene sites excavated by the FBMP. b. View of the excavation facing northeast. The site is located on the immediate periphery of collapsed blocks from the dismantling of the local cliff carved in the *Cabeças* (upper Devonian) Formation. In dotted line, the area recently excavated. c. Implantation of VPF site at the empty spaces left by blocks of sandstone that fell from the cliff.

**Fig 2:**
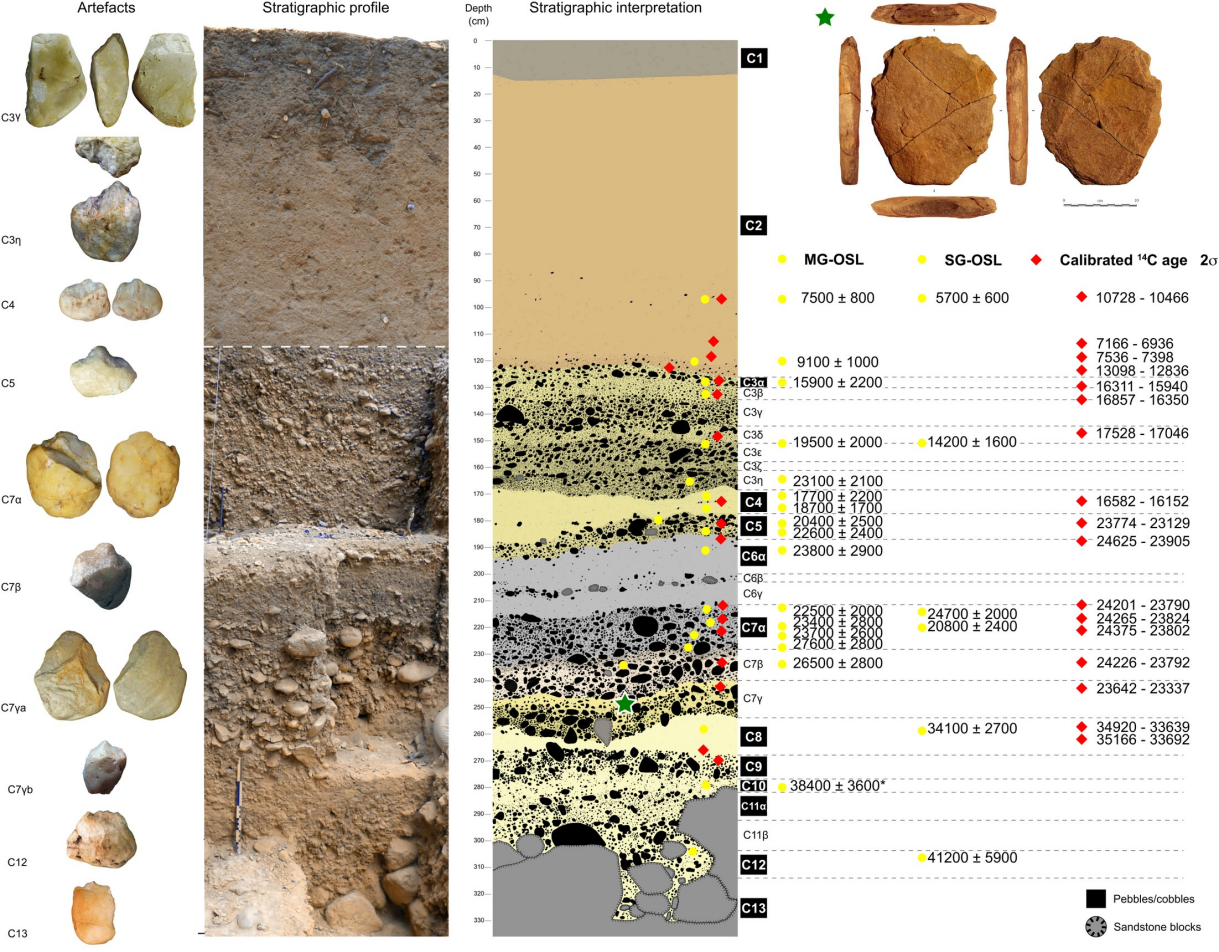
Vale da Pedra Furada, stratigraphic profile and synthetic stratigraphic interpretation. The red star marks the exact position of artefact here analysed within C7γa layer. (*) OSL date corresponding to C10 in a different sector than the rest of the stratigraphic profile. Artefacts are out of scale.

Since 2008, the Franco-Brazilian Mission of Piauí (FBMP) has undertaken interdisciplinary research on different archaeological sites inside and around the *Serra da Capivara* National Park (Brazilian Northeast) (Fig 1). Nine archaeological stratigraphic sequences and more than fifty archaeological levels attest to an important human occupation between 40 and 5 kyr BP [13, 16–18]. Ages were obtained both based on luminescence dating techniques (Single-grain or SG-OSL, and multi-grain or MG-OSL optically stimulated luminescence), and on radiocarbon measurements. All of the archaeological artefacts were manufactured mostly in quartz, quartzite, silicified sandstone, and silicified limestone. At the Vale da Pedra Furada archaeological site (VPF, do not confuse it with the neighbouring Boqueirão da Pedra Furada site [3–5, 24]), discovered by Gisele Felice in 1998 [30, 31] and extensively studied since 2011, the surface excavated until now is 20 m^2^, and the deepest geological level attained is located 270 cm below the actual soil surface (Fig 2). Twenty-two hundred lithic artefacts were found *in situ* in C7γ-a layer within a horizon of sandy-silt yellow/brown sediment stratified in a heterometric gradient composed by pebbles (Fig 2). One of the artefacts (N° 255660) made on a sandstone rock presents exceptional technical characteristics regarding the whole assemblage. This artefact is dated at the beginning of the LGM period.

## Materials and methods

### Materials

#### Geological settings, stratigraphy, and nomenclature system

*Geological settings and stratigraphy*. VPF is an open-air site on the left bank of the *Baixão da Pedra Furada* Valley that was formed in the empty spaces left by sandstones blocks, which fell from a cliff [32] (Fig 1C).

The Vale da Pedra Furada site (VPF) is related to two formal geological units [33]. The *Serra Grande* Formation (Silurian age; 150 m thick in the Pedra Furada valley), is formed by thick sandstones with cross-layering and quartz conglomerates, occasionally including also sandstone clasts derived from older units. The clasts (from pebbles to boulders) are not broken, they are well rounded and bladed [34]. Although the degree of selection is low, they have a grain-supported fabric [35]. Conglomerates and sandstones are well cemented by silica and hematite. The hardness of these rocks allows the existence of almost vertical cliffs. Above them are the *Pimenteiras* (Devonian) and *Cabeças* (upper Devonian) Formations, which are formed by layers of sandstone and siltstones. The first one is not well cemented and does not originate important reliefs, but the second one originates near-vertical slopes a major process that can be related to the LGM period.

The most important feature of the stratigraphy of the VPF site is the undulating vertical contact between well-defined layers, because the diameter of the coarse fraction obliterates the sharp contact.

In general, the sediments carrying the cultural remains are composed of quartz and quartzite blocks, boulders, cobbles and pebbles in a dominant matrix of sand and silt.

The clasts of the Quaternary conglomerate layers present very good rounding, a characteristic derived from the source in the Paleozoic sedimentary layers. The stratification of the layers is very well defined, with alternation of conglomerates and fine sediments beds. When observed from a short distance, it can be seen that the horizontality and sharpness of the contacts are obliterated by the presence of boulders, cobbles and pebbles, which cause the contact to be somewhat wavy at this centimetre scale. The VPF conglomerates are very poorly selected, no imbrication is observed nor normal or inverse gradation. We interpret that the main geological depositional process related to the formation of the site and the high concentration of blocks, boulders, pebbles and cobbles is lag deposition. The layers of fine sediments represent the kinetic energy of the environment in small colluvial cones of interannual rhythm, as demonstrated by the incipient development of soils. This type of sediments belongs to alluvial cones of tens to hundreds of meters wide that descended between the sandstone blocks derived from the *Pimenteiras* and *Cabeças* Formations. The yellowish-brown colour is interrupted by the accumulation of charcoal and soot also related to the human fire camps.

In eight sedimentary layers, it was possible to recognize successive episodes of more silt-sandy sedimentation (from bottom to top, C8/C6/C3/C2/C1) and moments where the gravel and block fraction dominate (C13/C12/C11/C10/C7/C5/C3) (Fig 2). The fine and coarse grain layers can be subdivided into lenses or also sub-units better related to an incipient soil development (C1/2 a and b), the partial laterization at the periphery of the sandstone blocks (C7 as subdivided into 5 subunits), the minimal movement of a fine brownish sediment derived from the weathering of basal sandstone blocks (C6), or a wash-over local phenomena taking away the fine fraction (lags). The presence of lag deposits explains that a very coarse sediment from boulders to cobbles represents actually only the energy to wash the fine sediments [9]. As for the C9/10/11/12 package, it alternates lenses rich in coarse elements (C9) with others where the fine fraction is dominant (C10, C12). Common to the site is the alternation between fine and coarser-grained sequences (e.g. C3). On the other hand, the concentration and diameter of the sandstone blocks is discontinuous between the layers. The C13 basal layer is composed of a large number of blocks that can weigh more than 100 kg. The C7 layer also has these blocks, but in lower concentration (weight around 10 kg). The level of rockslides, under the C6 layer, is the most massive of the sequence (Fig 2).

All these observations indicate a colluvium pattern, triggered by the gravitational rupture of sandstone blocks and the weathering of conglomerate components, added to the competence of runoff water from small waterfall located at the valley head, as observed today. That could explain the variation of the fine sediment supplies, depending on the characteristics of rainy periods. A few numbers of lenses show well sorted sediments that could be deposited by overflowing of the ephemeral streams (C6). In this type of sequences, it is important to understand that most of the time it is not represented by sediments, but by the hiatus between the layers. Therefore, the human occupation obviously occurred on a stable geological substrate. In this scenario, it was very likely that annual grasses covered the artefacts, protecting them from the next period of erosion-deposition, vegetation that left no visible geological mark, as it was interpreted in Monte Verde I site [9].

This particular discontinuous stratigraphic sequence with its micro-variations could reflect different climatic phases. The chronological results attribute a first set of layers to Pleistocene, i.e. between 41 and 14 kyr cal BP (C3 to C12) [18], and a second set to Holocene (C1 and C2) after 12 kyr cal BP (Fig 2).

The sedimentary characteristics of VPF site indicate, by their heterometric diversities and the laterization, the alternation of humid and less humid period, may be like today, in an interannual rhythm. The remains of extinct fauna recovered in other nearby sites dated to the Upper Pleistocene / Lower Holocene transition indicate a plant biome corresponding to a dry forest, consisting of a more or less continuous tree stratum and a grass cover that partially covers the ground [36]. This vegetation is closer to the *cerrado* (i.e. wooded park and gramineous-woody savanna) than to the actual *caatinga* (dry desert vegetation). This implies less dry climatic conditions than the present ones [37–41].

**Fig 3:**
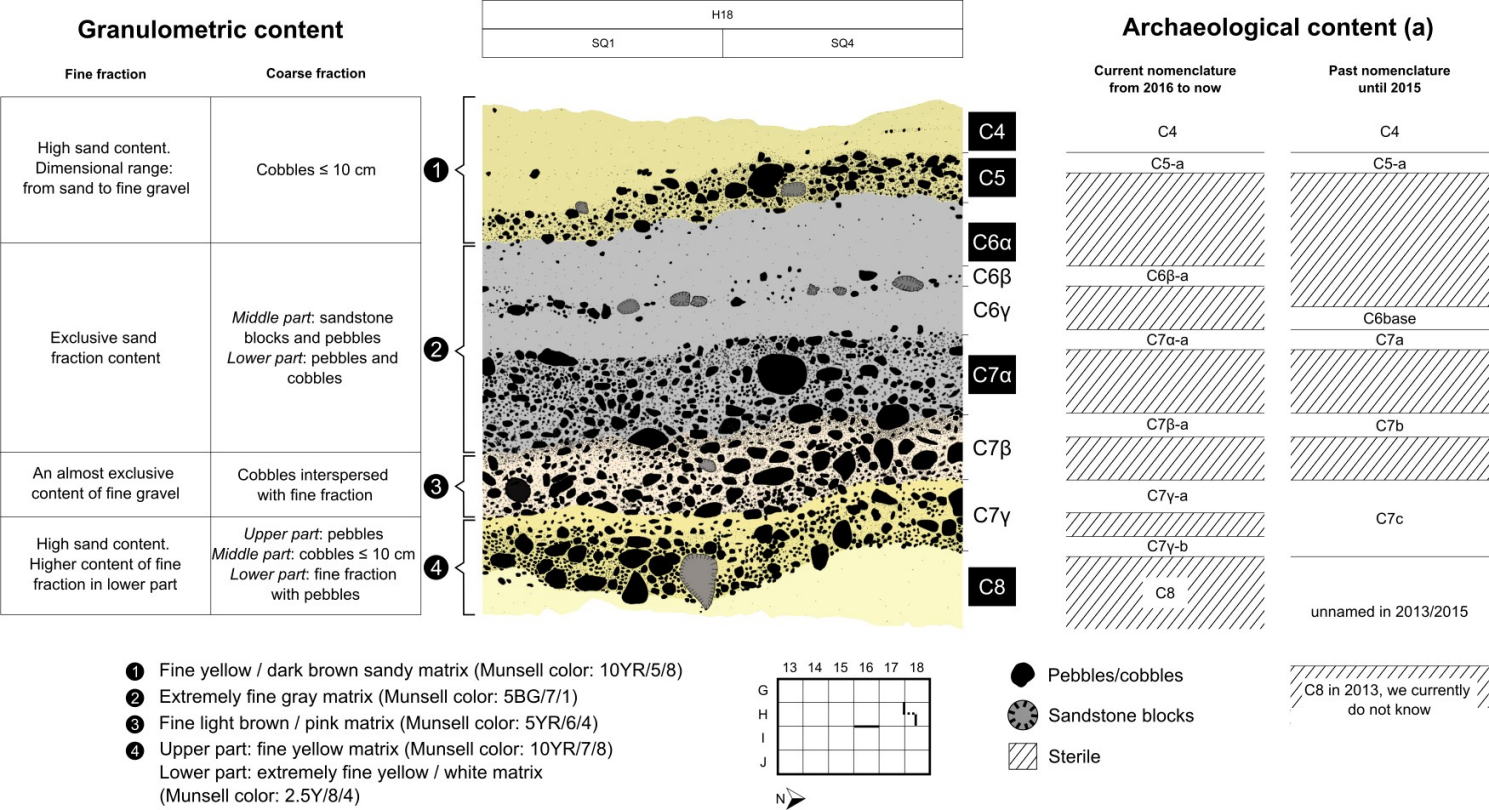
Detail of the granulometric and archaeological criteria taken into account to construct the chronostratigraphy of Vale da Pedra Furada.

*Nomenclature system and chronostratigraphic construction*. The nomenclature system used to construct the chronostratigraphy of Vale da Pedra Furada has been applied systematically since the start of the excavations. Previous publications [14, 18] summarize the chronostratigraphic study. We present here the details of the nomenclature system used, in order to dispel any doubts about our understanding of the site’s stratigraphy and the integrity of the archaeological horizons identified. Fig 3 shows an example of the analytical study performed.

To obtain a high resolution chronostratigraphic understanding, taking into account the particle size and colorimetric composition of the sediments, we make a first distinction based on a nomenclature using Arabic numbers (1, 2, etc.), then if necessary we make a subdivision to determine subsets, and add a nomenclature using Greek letters (“alpha”, “beta”, etc.). Finally, to designate the presence of an archaeological horizon we use another name, using the Latin alphabet (a, b, c, etc.).

For presentation purposes and according to knowledge of stratigraphy, in previous articles prior to 2015 [14, 18], we have mentioned archaeological layers only through the words "a", "b", "c ". For these reasons, there is a change of name between these articles and the present one. For example, what in Boëda et al. [14] was called “C7a”, since 2016 is actually “C7α-a”; and so on for “C7b” which is in reality “C7 β-a”. The same with “C7c” which groups together two different archaeological horizons: “C7 γ-a and C7γ-b”.

Since the 2016 mission, the excavation area has been significantly enlarged at VPF, both horizontally and vertically. The visualization of the stratigraphic over more than 4 m long and 2 m wide made it possible to determine the sedimentary units underlying the C7c layer established in 2013, which, let us recall, became C7γ-a and b). A net and clear dilation of the stratigraphy made it possible to bring to light a succession of sedimentary layers respectively called C8/9/10/11/12. This raises the problem of the old name C8 established in 2013. For the moment we prefer to remain cautious because we do not have a visual tracking of the stratigraphy between the C8 of 2013 and the current C8. However, in a sedimentary aspect they are completely different and therefore cannot be named identically. What we know for sure is is the C8 of 2013 is older than the current C10, if not older.

The same process occurred with layer C6, which in the initial excavation only suggested an archaeological horizon named C6base in contact with C7a (established in 2013) which has since become C7α-a. The aforementioned opening revealed significant microstratigraphy within C6, differentiating between alpha, beta and gamma horizons, with the presence of lithic artefacts in the C7beta subdivision, then named C7 β-a.

Considering the reasons explained above, we can say that this is not really a nomenclature change, as it has always been the same. Only the way of presenting it in publications has changed. Therefore, no modification to the chronostratigraphic construction was made.

### Archaeological context of the piece

**Fig 4:**
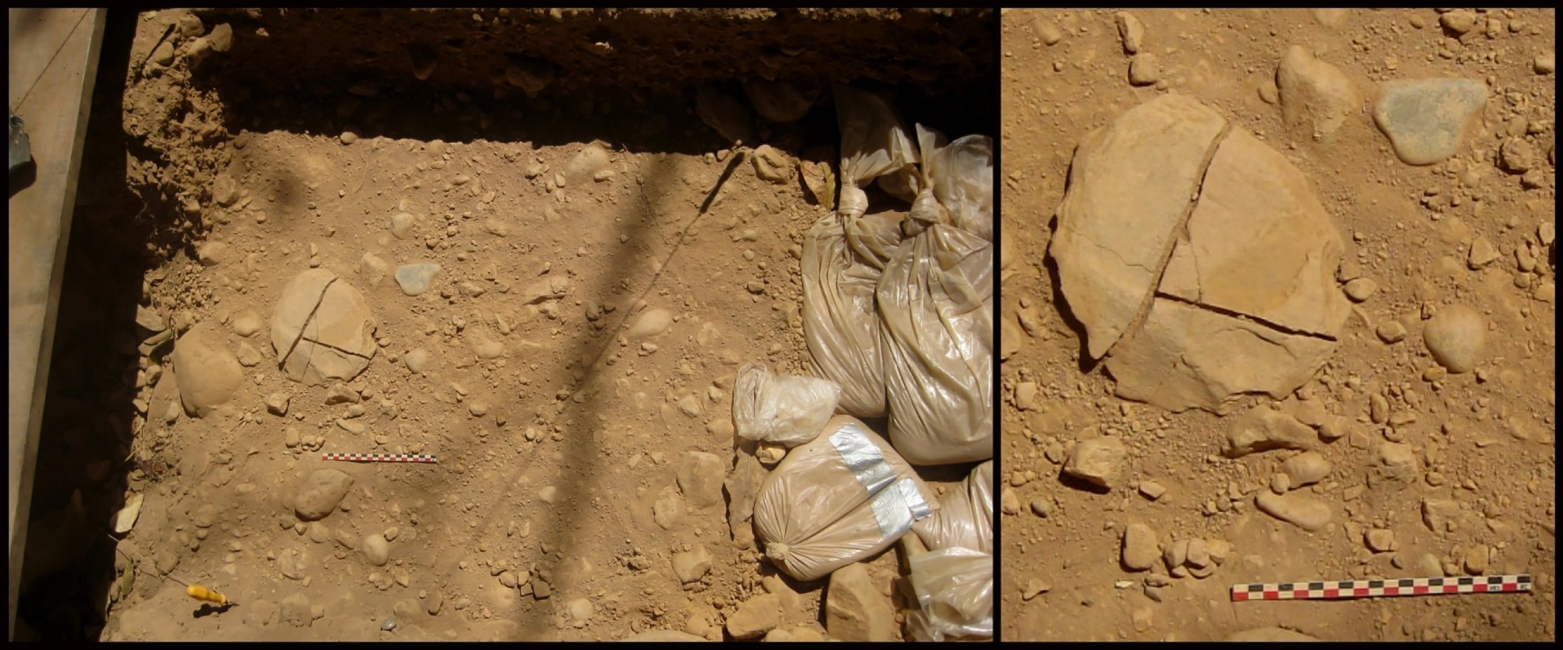
Archaeological context of the piece. Layer C7γ-a with artefact n° 255660 found *in situ*.

C7γ-a layer contained an entirely unexpected artefact, making an exception with its raw material used, its size, its volume and its silhouette. It was found lying flat, fractured, *in situ* (Fig 4). We note that the platy artefact, with its long and intermediate axes, was already parallel to the surface of a sandy lens. It has three fractures. Two old T-shaped fragments, subdividing the piece into three fragments, which can be fitted visually. The breakage is old but clearly on the edge. The patina observed on the faces of the fractures is slightly different from the patina visible in the rest of the piece, but it is clear that the fractures are not recent. The third fragment broke off after the discovery of the piece, during the release of its sedimentary matrix.

### Methods

#### Excavation method

The excavation method has been consistently the same since the beginning of archaeological investigations at the site. The excavation technique pursued a systematic three-dimensional recovery of all lithic artefacts (knapped or not) greater than 2 cm, following the geological stratigraphy. The location of non-anthropic objects was logged by quarter-metre in 50 mm thick spits. Systematic sieving to 2 mm enabled recovery of abundant small fragments of knapping debris and retouch flakes. Three-dimensional coordinates were recorded for each charcoal sample. The record and recovery of the artefact N° 255660 was carried out through the use of nitrile gloves without powder, in order not to contaminate the piece with hand grease. The piece was immediately preserved along with a sample of its sedimentary matrix, in order to carry out future micro-remains analysis.

#### ^14^C analysis

Eighteen charcoals have been dated in the C2 to C8 layers: three out of the C2 layer, one charcoal at the C2/C3 layers contact, three out of the C3, one out of the C4 layer, one out the C5 layer, one at the C5/C6 contact, three out of the C6 layer, three out of the C7 layer and two out of the C8 layer.

Samples were treated at LSCE, Gif-sur-Yvette (Gif- and GifA- prefixes as chemistry lab identification), according to a classical chemical treatment in, successively, HCl 1.2N, NaOH 0.1N, and then HCl 1.2N with rinsing between each step with ultrapure water.

Prior 2015, physical measurement was handled by the LMC14 (SacA- prefix as measurement lab identification). Clean samples were then transformed into CO_2_ thanks to CuO as source of oxygen in quartz tube sealed under vacuum and left for one night at 650°C. Evolved CO_2_ is dried, measured, transferred in quartz tube and then sealed in a glass line kept under 10^−5^ Torr vacuum. Reduction to C graphite and physical measurement were then performed by the LMC14, Saclay [42]. CO_2_ was converted to graphite using the hydrogen reduction method with iron as catalyst prior AMS measurement.

After 2015, physical measurement was handled by LSCE (ECHo-prefix as measurement lab identification). Clean samples were successively converted into CO_2_ and reduced in C in presence of H_2_, using an Automated Graphitisation Equipment AGE3 [43]. Pure graphite is then pressed in presence of ultrapure iron into a target to be introduced in the solid source of *ECHo*MICADAS [44], a Compact Radiocarbon System [45] able to run very small samples.

In both cases, the ^14^C/^12^C and ^13^C/^12^C ratios were measured by Accelerator Mass Spectrometry (AMS), and were background-corrected and normalized to the HOXII standard. The ^14^C ages were corrected for isotope fractionation using the AMS or MICADAS measured ^13^C/^12^C, which accounts for both natural and machine fractionations. ^14^C data are presented as conventional ^14^C age calculated using the Libby half-life of 5568 years following the methods of Stuiver and Polach [46]. They are associated to one standard deviation. Calibrated ^14^C dating derives from SHCal20 [47] using OxCal software (v.4.3.2, Bronk Ramsey 2017), two sigma of calibration intervals (95.4%) are reported in S1 Table in S1 File.

#### OSL analysis

Eighteen sediment samples have been dated and are presented in S2–S4 Tables in S1 File. Some of them have already been published [14, 18] and some are unpublished results. Some sediments were sampled by inserting PVC tubes in the stratigraphic profiles. Others were collected by scraping the sediment under controlled lighting conditions (red light, during the night). Some level attributions have been revised between Lahaye et al. [18] and the present paper, linked with the progress of field operation and a better understanding of the global site configuration (see section Nomenclature system and chronostratigraphic construction, and Fig 3).

OSL ages were obtained from quartz fractions extracted from the sediment samples, after mechanical and chemical treatments (see, for example, for methods commonly used in CRP2A laboratory: [17, 18]). Methods used to determine the equivalent Dose (D_e_) using single-aliquot regenerative dose protocol (SAR) [48–50] and applying the Central Age Model (CAM [51]) or the BaSar approach [52, 53] were detailed in Lahaye et al. [18]. Similarly, the measurements and methods used to determine the internal and external dose rates were detailed elsewhere [18]. S2 and S3 Tables in S1 File provide the characteristics of the D_e_ measurements, respectively for multi-grain and single-grain analysis, and the ages. S4 Table in S1 File shows the internal and external radioelement contents for all samples.

The new OSL ages presented here confirm the previous papers conclusions: multi-grain and single-grain ages, obtained for the same sample (where applicable), are in good agreement, within uncertainties. OSL and ^14^C ages are also in good agreement with each other, in the large majority of cases, and stratigraphic order is respected by both ^14^C and OSL ages sequences.

#### Chronological model

A hierarchical Bayesian chronological model was constructed, based on ^14^C ages, OSL ages, and stratigraphic information. The Bayesian age-modelling software package ChronoModel has been used (v.2.0.18 [54, 55]) ^14^C calibration has been done using SHCal20 [47].

MCMC parameters were as follows: investigated study period: 82,000–1,000 BP; MCMC setting: 3 chains, 1000 burn-in iterations, adaptation on max. 20 batches of 2000 iterations each, acquisition on 100,000 iterations with a thinning of 10.

The model is presented in S1 Fig (events in a, and phases in b) in S1 File, and results of the model are shown in S2 Fig and S5 Table in S1 File, showing the phases ages with an 80% confidence level.

#### Stone tool scanning

The 3D model of the artefact N° 255660 was made by photogrammetry. For the shooting, we used a camera Canon EOS 6D, a lens Canon EF 100 mm 1:2.8 L IS USM, an automatic turntable, a set of 12 bits coded targets, a tripod and a light box Havox HBP-40XD, with 6 dimmable LED bar with a 93 CRI. We generated the 3D model with the software Agifot Metashape v.1.6.2. We used a classical workflow for close-range photogrammetric modelling using the following parameters in Metashape: first, with the alignment in high accuracy of the set of 179 photos (point cloud of 910 609 tie points); second, the building of a dense cloud in high quality in mild filtering mode (dense cloud of 18 479 004 points); third, the building of a mesh in high quality (mesh of 3 965 800 faces), the scaling with 12 bits targets (average error of 0.48 mm) and then the building of the texture in the adaptative orthophoto mapping mode with a 4096 texture size/count. The video used the high-resolution model, the PDF3D attached to the paper shows a 3D model obtained with the high-resolution model with a decimation of 300 k faces and a generation of another low-resolution texture for optimizing the weight of the PDF3D file.

#### Techno-functional and traceological analysis

The analysis of the artefact N° 255660 was subjected to a large techno-functional analysis [56] focused on the observation and recording of the structural characteristics of the cutting edges and all modified and natural surface present in the specimen. After this structural characterization, a two-step procedure was carried out. Firstly, we analysed the surface conditions of the ribs’ distal parts participating in the development of the bevels. Secondly, we proceeded to observe the surface states of the edges. Then, different surface states were identified, which permitted the elimination of possible taphonomic alterations in an immediate manner. Finally, these data were crossed in order to highlight the different technical and functional moments. After all this techno-functional analysis, the data obtained by the traceological analysis was integrated into the interpretation of the artefact.

*Observation and recording of structural characteristics of modified and natural surfaces*. This stage consists of a topographical analysis of the artefact. The features (traces) present on the surfaces of the artefact were observed and recorded. These traces are either the result of anthropogenic actions (negative removal) or natural (fracturing zones corresponding to the bedding of the plate). A first classification threshold was achieved between the techno-functional consequences of the negatives of removals and the different natural areas of the artefact. The techno-functional consequences (*sensu* [56]) encompass the effects produced by the three technical operations recognized so far: affordance, debitage and shaping. In this case, two operations are present: affordance and shaping.

Affordance refers to the selection of techno-functional criteria naturally present in the initial block and which will be maintained and present in the final product. These natural areas participate in the functionalization of the piece. Shaping refers to the configuration of a piece, inside a mass of material that is worked from the start to obtain techno-functional criteria that are not found in the initial volume [56–58].

These two technical operations can produce two types of consequences on the piece: technical consequences (concave, convex, flat surfaces, backs, etc.) and functional consequences (cutting edge, cutting wedge, cutting wedge angle, rake and flank face, etc.).

The recording of these techno-functional observations was carried out through diacritical diagrams [59, 60], 3D modelling and high quality photographs.

*Observation of the surface condition of the ribs*. The previous techno-functional reading required a second stage of analyses, given the particular characteristics of the piece N° 255660. We have indeed observed different surface conditions on the ribs (the lateral ribs of each negative of removals, as well as the ribs that participate in the structures of the cutting edges). In particular, the state of differential abrasion in certain areas with strong techno-functional modification has led us to systematize the recording of this information through computerized topographic diagrams guided by naked-eye observation and with a binocular magnifying loupe (40x to 80x). This made possible to locate the concentrations of abraded areas, to classify them qualitatively and to determine a second classification threshold between modifications caused by taphonomic agents and anthropogenic modifications.

*Definitions of techno-functional moments / stages*. The crossing of techno-functional data (technical consequences on each surface and those corresponding to the state of abrasion of the ribs) allowed us to reach a third classification threshold: the moments / stages of transformation of the part. The computerized superposition data, in different thematic layers, allowed us to observe patterns of modification in the piece. On the basis of these patterns, the stages of transformation were induced.

*Traceological analysis*. A Leica MZ16 A stereomicroscope, a Leica DM 2500 M metallurgical upright microscope and an Olympus BH50 metallurgical upright microscope equipped with Nomarski prisms, were used. Determination of use-wear on this artefact was done by following the parameters defined during preceding studies of quartz [61, 62], and by referring to our own databases of quartz and other heterogeneous rocks such as rhyolite and quartzite [63, 64].

## Results

### Archaeological horizons at the Vale da Pedra Furada site and others artefacts in C7 γ-a

Throughout the VPF stratigraphic sequence, there are fifteen distinct archaeological horizons separated by sterile sedimentary layers. The artefacts are mainly lithic specimens, due to the acidity of the soil. The traceological analysis carried out on a large number of artefacts attest the presence of cutting edges with or without or retouch which were in contact with animal and vegetable materials (woody and non-woody plants). The dominant raw materials used throughout the sequence are quartz and quartzite. Only the best qualities of raw material are selected. If the use of quartz is generally dominant, there is a variability from the oldest to the most recent levels. The oldest levels (C13 to C7γ) present a large percentage of quartzite tools, while the use of quartzite remains limited. We can put in parallel a second observation. When the use of quartzite is rare, it is reserved for large tools, while when there is an equality in the use of quartzite and quartz they participate in the manufacture of the same tools. In the oldest levels, the most massive tools are made on pebbles of quartz and no longer of quartzite. A third raw material, sandstone, is widely used in layers C13 to C7γ. Different qualities are used depending on the target tool type. In general, these are objects that have been used because of their abrasive surfaces. There are active tools of small sizes and passive tools. A special quality so-called silty sandstone was used in the manufacture of the artefact N° 255660. The acquisition of these raw materials could have been done in the immediate vicinity of the site, within an approximate radius of a hundred meters. On the taphonomic aspect of these industries, we refer to the studies published on the subject [14, 23].

On the technical and morphological level, we can differentiate several categories of tools. The most classic are three in number: *rostrum* (delineation break to externalize a linear or micro-denticulate active part), *bec* (delineation break to exteriorize a point less than 6 mm in length), and *single bevel* (*biseau simple*). Depending on the layer concerned, we classified: denticulate, scrapers, side-scrapers, perforators and bifacial converging tools (see S4–S10 Figs in S1 File).

There are two types of production: debitage and shaping. Thereafter, depending on the target type of delineation, retouching is necessary or not. Under the term debitage we must distinguish several modalities. The first, which is the most recognizable but not the most frequent, except for the most recent Pleistocene levels, is bipolar percussion. The second is the opening of a striking platform to produce a small series of 2 to 3 flakes. This technique is found exclusively in the archaeological horizons underlying the C8 sedimentary layer. The third and the most original modality covers (a) a selection phase of a particular pebble techno-type, and (b) a shaping phase of the future tool by the systematic production of the same flake morpho-type. This is clear evidence of predetermination, because a large part of the technical characters of this flake are known in advance. This flake, recognizable among all, is subsequently the preferred support for a large number of tools. This technique is observed from the archaeological horizons C7a and C7b to become omnipresent in the following archaeological horizons and in particular in those of C3. Shaping is present in all the archaeological horizons, but little represented in lower levels (C9 to C13).

**Fig 5:**
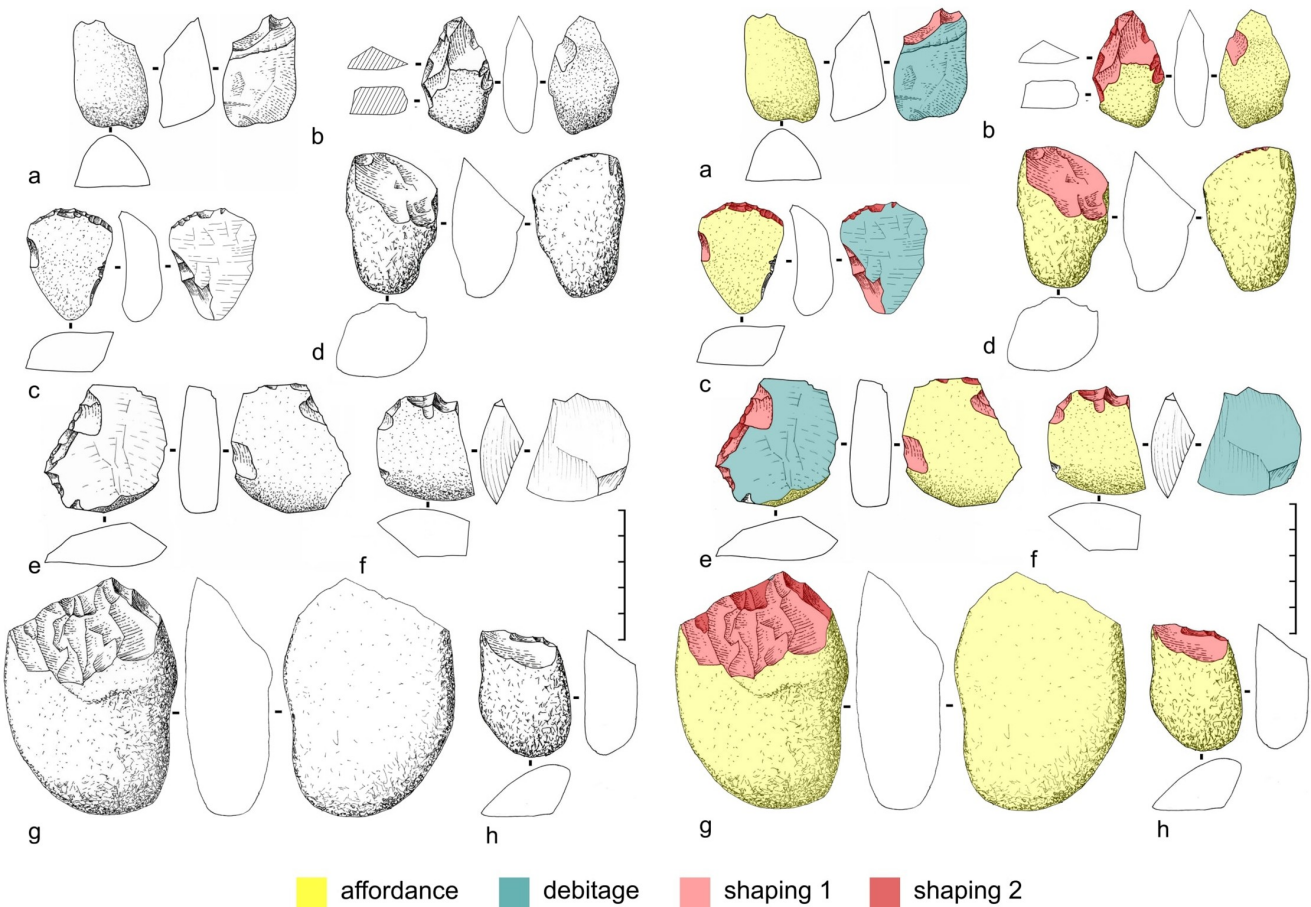
General artefacts of the C7γ-a layer. (a) *bec*-type cutting-edge on split flake from quartz pebble, (b) bifacial symmetrical convergence type cutting-edge on quartz pebble, (c) end scraper-type cutting-edge on unipolar quartz flake, (d, h) simple bevel-type cutting-edge on quartz pebble, (e) side scraper with inverse retouch on quartz pebble flake, (f) *bec*-type cutting-edge on quartz pebble flake, (g) unifacial asymmetric convergence type cutting-edge on quartz pebble.

**Fig 6:**
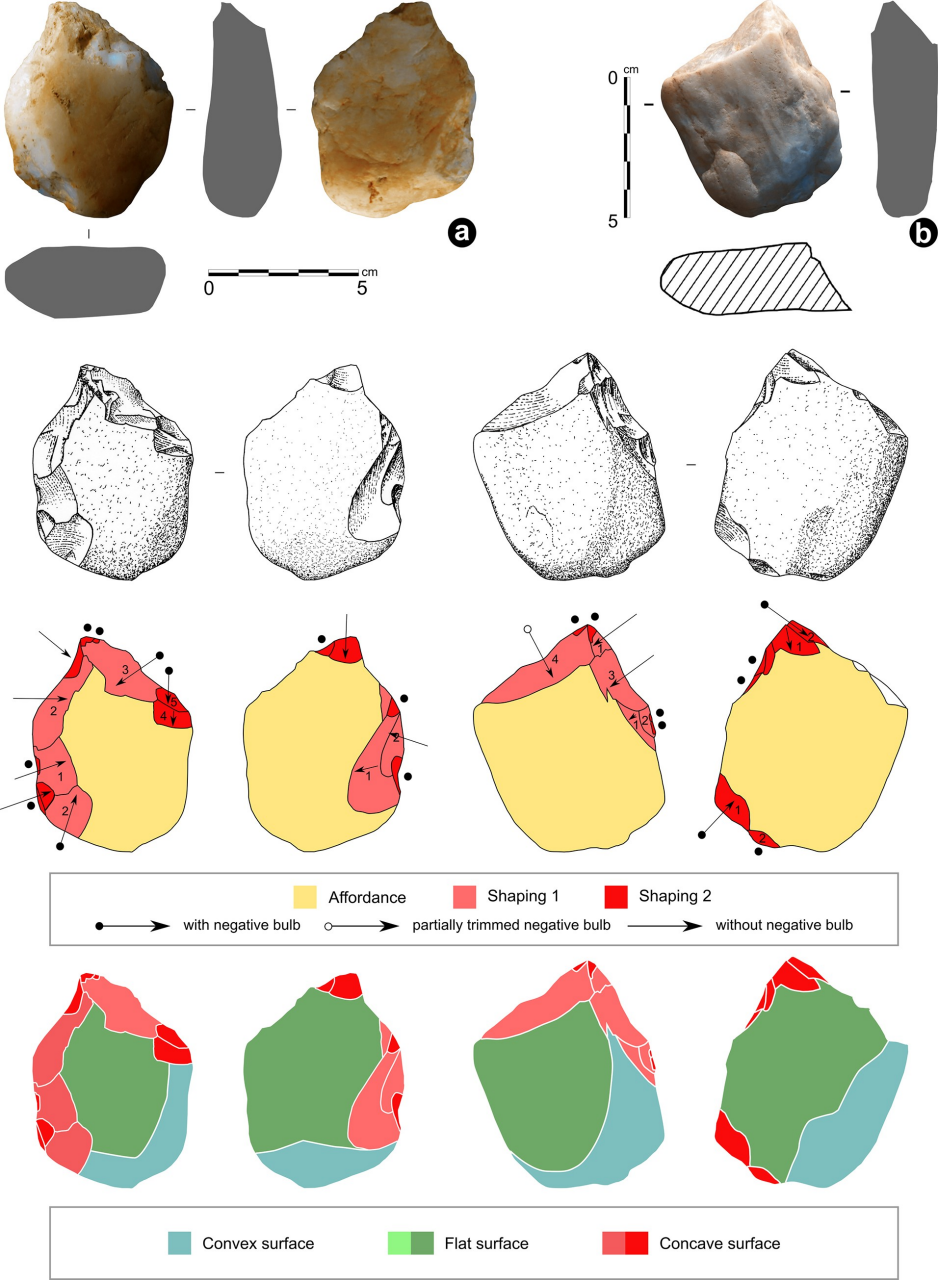
VPF archaeological site. Bifacial asymmetric convergence cutting-edge type on quartz pebble from C7γ-a layer: (a) FUMDHAM code: 257350; (b) FUMDHAM code: 257213. From top to bottom: Photographs, conventional drawings, diacritical analysis, and techno-functional analysis.

**Fig 7:**
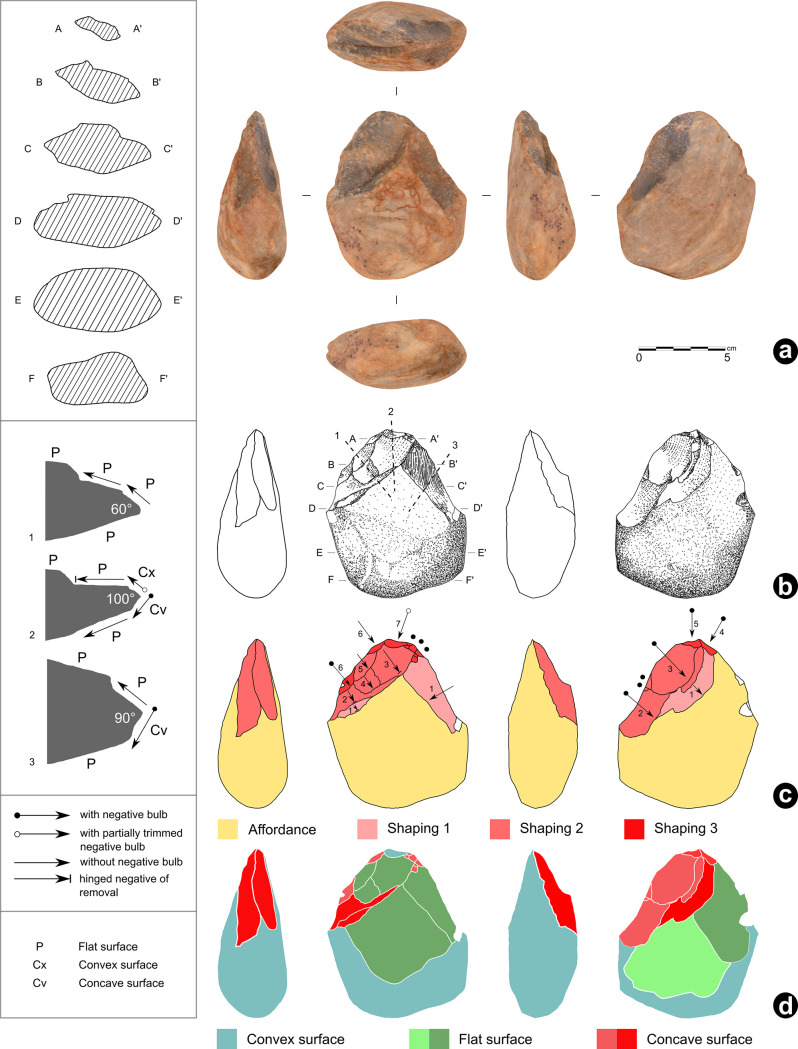
VPF archaeological site. Bifacial symmetric convergence cutting-edge type on quartz pebble from C7γ-a layer. FUMDHAM code: 255690. (a) Photograph of the six standard views, (b) conventional drawing, (c) diacritical analysis, (d) techno-functional analysis.

C7γ-a is an archaeological horizon which is characterised by the presence of other tool techno-types in greater quantity (Fig 5). These are tools on quartz and quartzite, but, for the first time, the presence of convergent and bifacial shaped pieces (Figs 6A, 6B and 7A). The detailed techno-functional analysis of these pieces (Figs 6, 7C and 7D) makes it clear that target objective is a convergence of edges releasing a point, which in the three cases is fractured. Another tool type presents all bifacial typical characteristics, but we cannot qualify it as bifacial *sensu stricto*. It is a very enigmatic piece, because given in a Holocene context it would possibly have been taken as a projectile point preform (Fig 5B).

In summary, Vale da Pedra Furada archaeological sequence, through its various sedimentary deposition modes, allows for the first time to have a clear dated chronostratigraphy. The archaeological horizons, undoubtedly Pleistocene, indicate clear technical differences in the qualitative characteristic of lithic industries.

In other words, we have enough arguments to stop considering these Pleistocene industries as a stable and repetitive in time. On the contrary, the presence and/or absence of certain techno-type tools, different functioning modes, and different management of resources show that this period is crossed by very different technical and cultural trends.

### C7 chronology and composition

The layer C7 is between two layers composed by sandy-silt sediments without cultural remains (C6γ and C8) and is composed by three distinct sedimentary subsets (lenses α, β and γ), corresponding to an alternation of fine and coarse deposits that have undergone fine grain washing phenomena, oxidation, and laterization. The sub-layer C7γ, at a depth of ~2.50 m from the surface, has a heterometric gradient from a bed of pebbles at its base to a dominance of fine sandy-silt sediment at the top. In a matrix supported sediment, an archaeological horizon called C7γ-a was individualized. It was excavated on 6 m^2^. It yielded 2200 artefacts over 1 cm in size.

The major part of the ages for the VPF site have been published elsewhere [14, 18]. The ages are in stratigraphic order, and in good agreement between OSL and radiocarbon, all over the sequence. As far as C7 level is concerned by the present study, it has been dated using four sediment samples (all four dated by MG-OSL, and two of them also dated by SG-OSL), three located in C7α and one in C7β, and also radiocarbon dates on seven charcoal fragments.

OSL results place all dated C7 sub-levels between around 27 and 21 kyr cal BP (S2–S4 Tables in S1 File) in agreement with ^14^C outputs that cover the 23.4 to 24.5 cal kyr BP range [14, 18] (S1 Table in S1 File). The hierarchical Bayesian chronological model constructed based on ^14^C ages, OSL ages and stratigraphic information (S1 and S2 Tables in S1 File) place C7γ phase between 27.6 and 24.0 cal kyr BP (S5 Table in S1 File).

### Techno-functional description of artefact N° 255660

**Fig 8:**
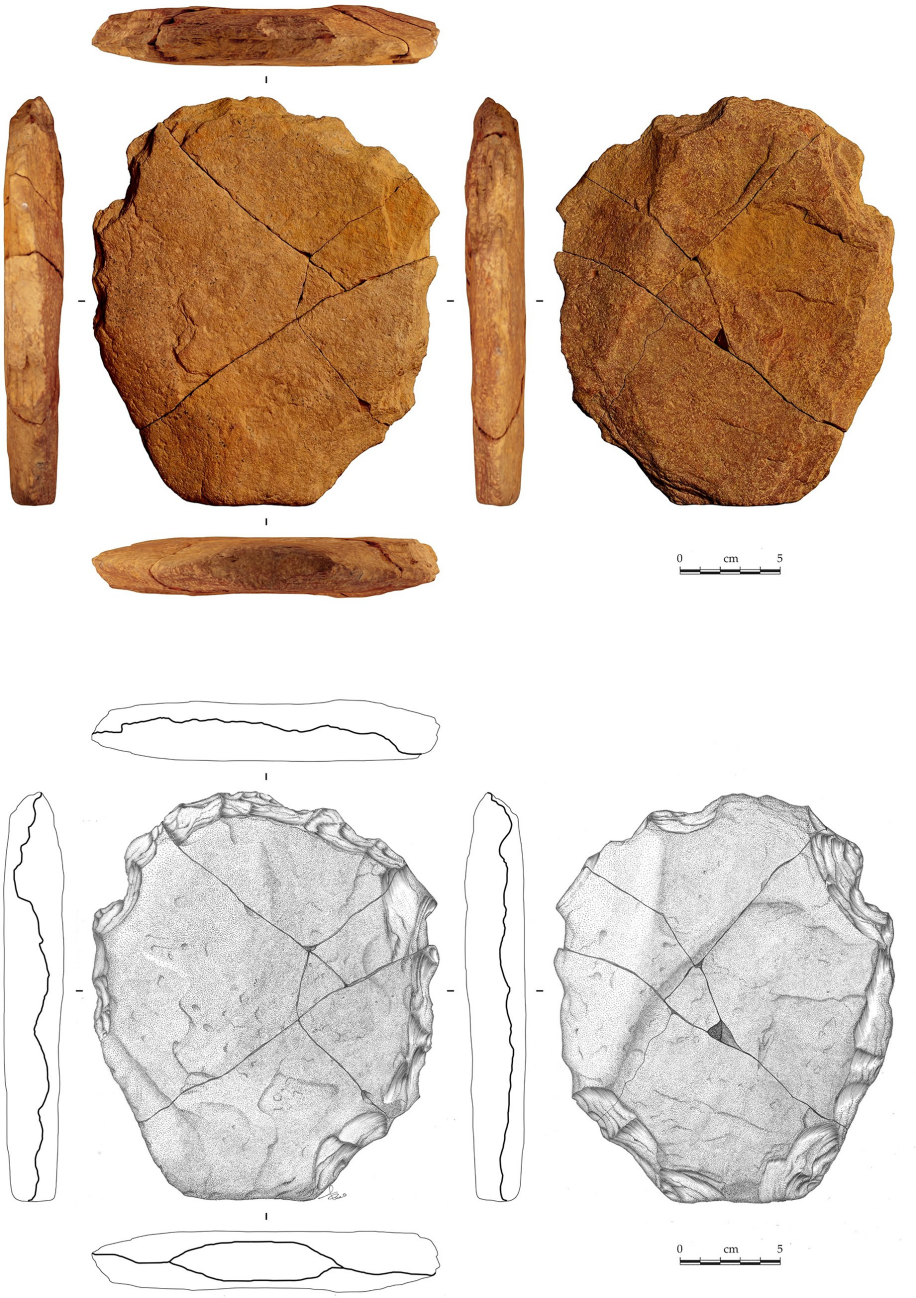
VPF shaped silty sandstone plate (artefact N° 255660). See S3 Fig in S1 File for a 3D scan of this artefact.

It is a 7.5YR/6/6 orange well-cemented (or silicified) silty sandstone plate 21.0 cm long, 18.5 cm wide and 2.9 cm thick (all maximum measurements), with two parallel faces (Fig 8).

**Fig 9:**
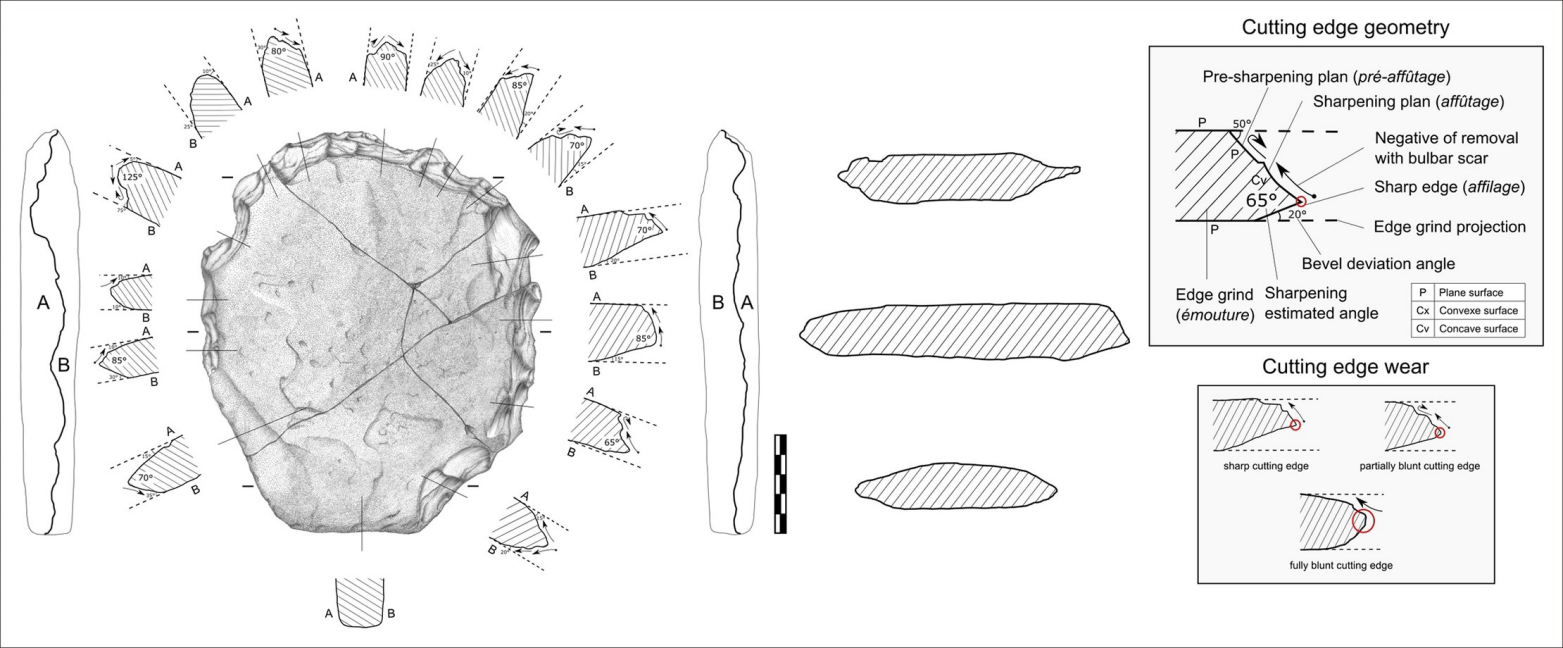
Cutting edges geometry and artefact’s sections. The techno-functional study determined the presence of three types of cutting edge wear: fresh, partial and fully blunt.

The surfaces are not completely regular given the presence of inclined sides. The general shape is hexagonal, symmetrical in its maximum length, and it can be divided into two subsets. The first has the silhouette of an isosceles trapezoid made of rectilinear edges, while the second has a quadrangular silhouette including two parallel edges and a semi-circular transverse edge with a macro-denticulate delineation produced by large adjacent notches following an internal percussion with a hard mineral hammerstone. No such flakes were found during the excavation. The section indicates a double bevel configuration on the entire periphery with the exception of the short side of the trapezoid which has a U-shaped section. The angle of the bevels oscillates between 65° and 85° (Fig 9).

**Fig 10:**
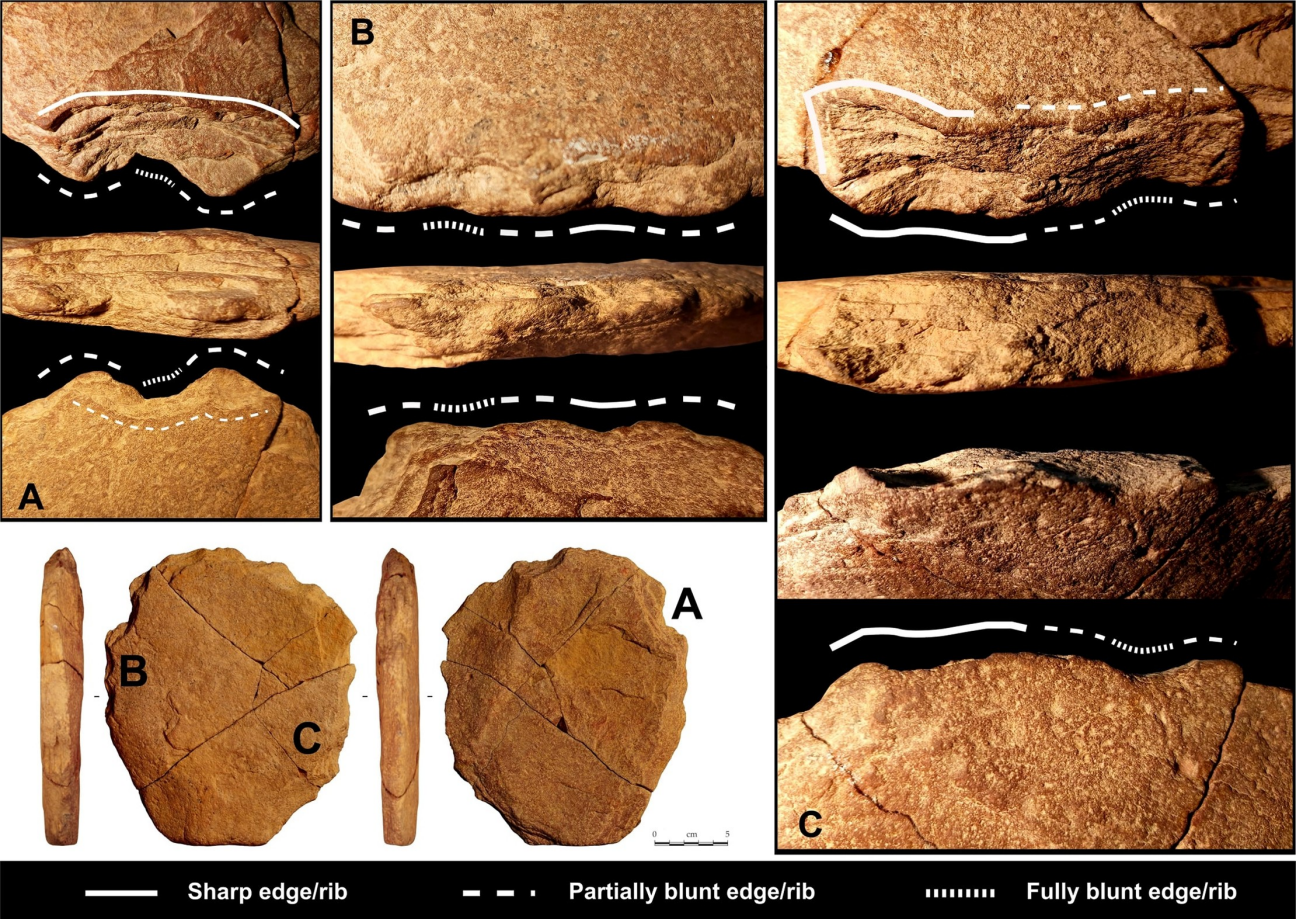
Macro-trace analysis of the artefact n° 255660.

The surface condition of the cutting edges varies from fresh to fully blunt, predominating the partially blunt wear (Fig 10).

**Fig 11:**
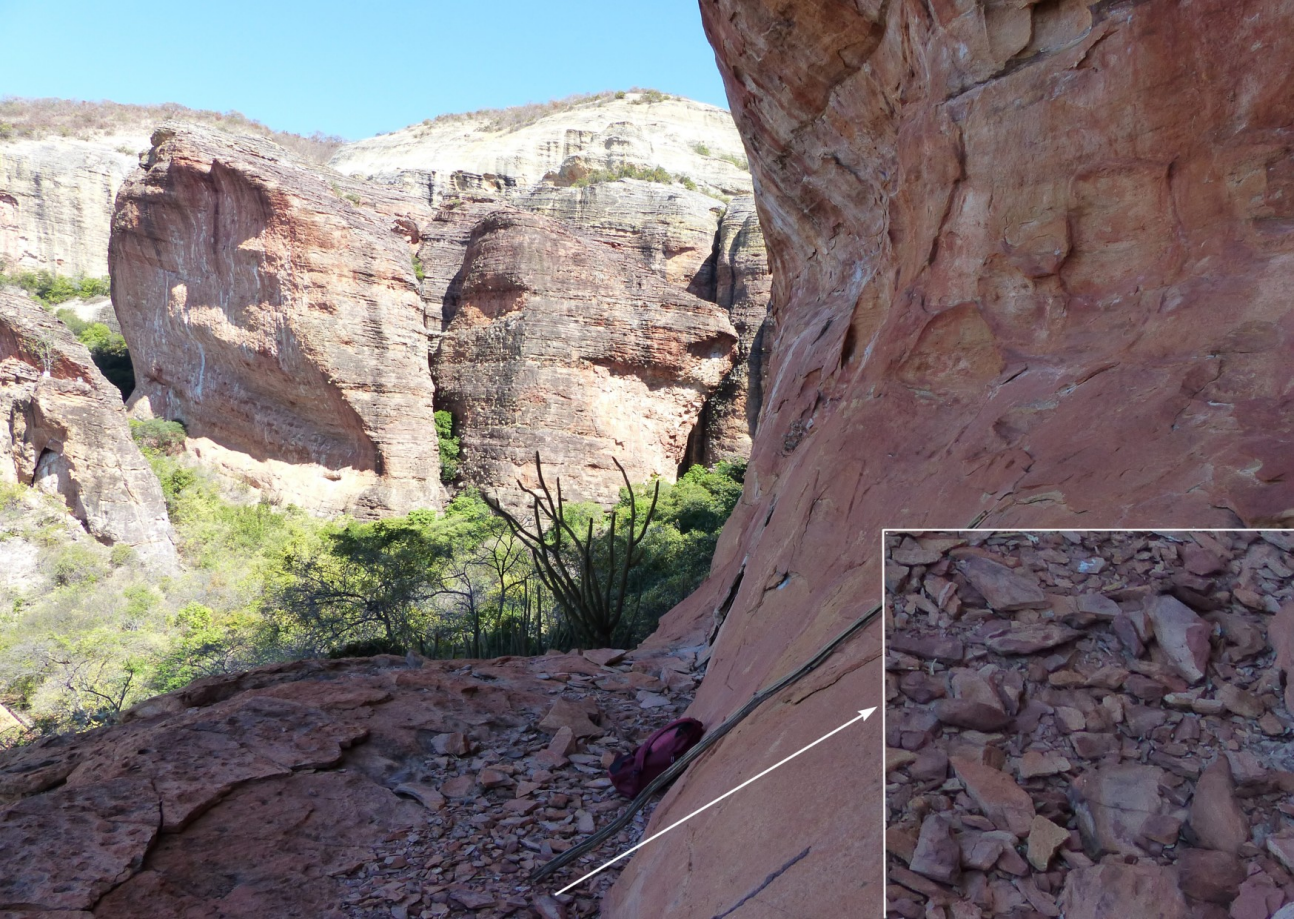
Structural flat of the cliff, located 100 m from VPF site and more than 30 m over the valley floor. In the zoomed image the sandstone plates that may have served as tool blanks.

The raw material used outcrop in the upper Devonian *Cabeças* Formation, in a 10 cm thick layer, approximately. A silty sandstone, similar to the raw material of the artefact N° 255660, was found on a small structural flat of the cliff a hundred meters from the site and more than 30 meters over the valley floor (Fig 11).

**Fig 12:**
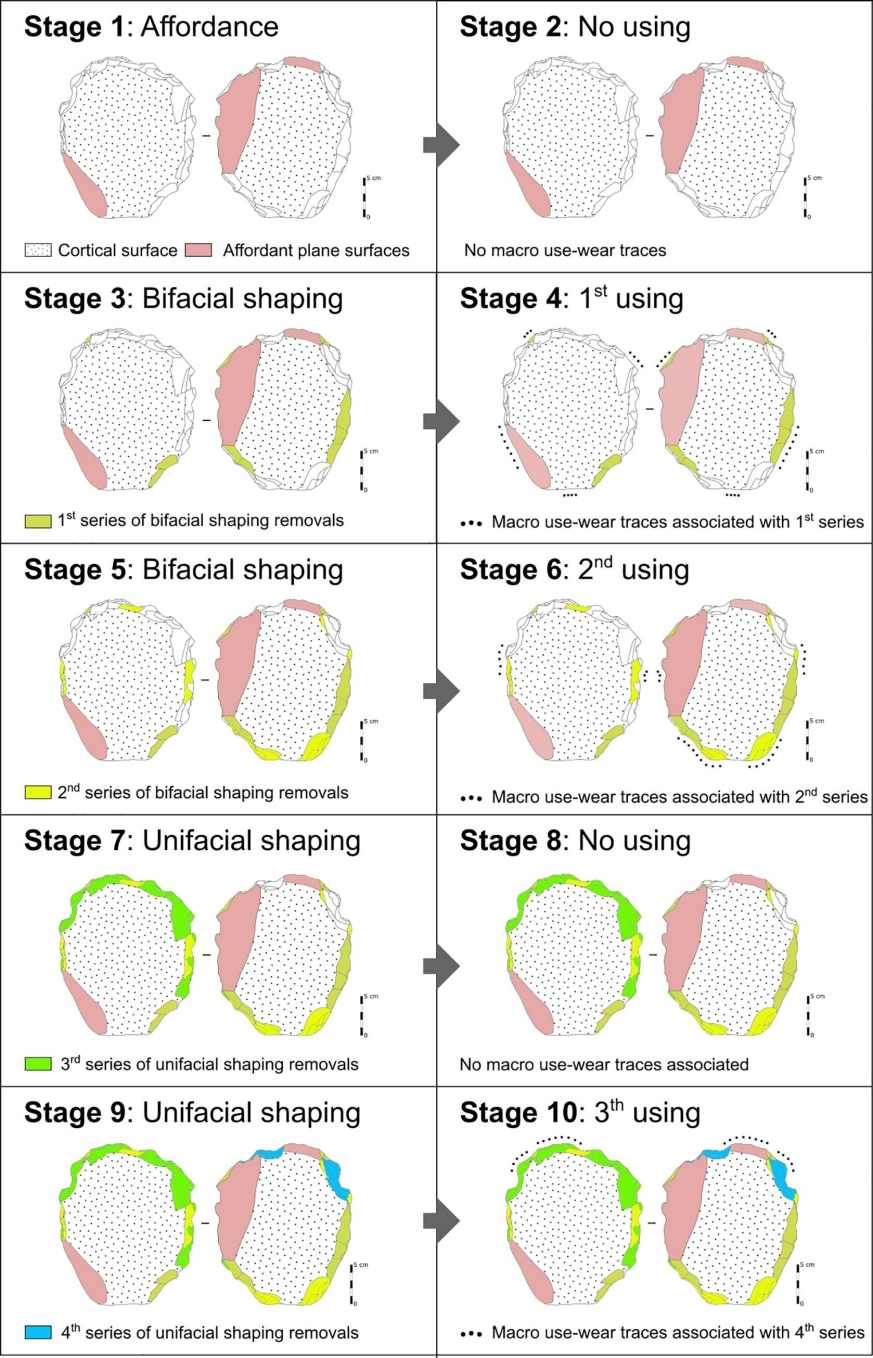
Technological analysis of the artefact n° 255660.

We distinguish five stages of transformation leading to the final volume and three moments of edge alteration (Fig 12). The **first stage** corresponds to the affordance: selection of plate thickness, raw material quality and natural inclined planes on two surfaces. The **second stage** is attested by distal ribs of very blunt removals scattered around the periphery. They indicate that the trapezoidal and quadrangular areas are largely elaborated, while the denticulate delineation is not yet present. The trapezoid area evokes a possible peduncle for hafting. The **third stage** relates to blunt distal ribs of less importance than the previous ones. The new removals reaffirm the quadrangular form deductible from the previous stage and install new bevels with partially blunt edges. The trapezoidal area is reused after “repair/maintenance” and thus the peduncle role is confirmed, given the over-erosion of the ribs and the maintenance of the specificity of its flat and U-shaped section. This peduncle is extended by parallel opposite edges, both with small unifacial notches with edge wear creating a rounded encroachment. Probably, these elements participated in a hafting method. The quadrangular area presents also wear on ribs and edges in the form of abrasion traces, therefore a violent action on a hard material is unlikely. The **fourth stage** presents a series of large adjacent notches on the quadrangular area creating an arc opposite the peduncle, respecting some old edges. Ribs’ surfaces are fresh. There is therefore no reduction in dimensions. A complete silhouette appears for the first time. No macro-trace of alteration is present on the new cutting edges, which questions the finality of this new configuration stage (Fig 12). Since the existence of a previous macro-denticulation is not certain, probably a new silhouette was developed, meaning either a new function or a simple repair. If it is the second case, the notches should be of the same dimension and there should necessarily be a reduction in the surface. This is contradicted by the presence of a section of invariable thickness and removal negatives with very blunt ribs and edges from the second stage. From a functional perspective, the probable peduncle (keeping the same function and the same use), is opposed to an almost sub-circular part, devoid of any post-fabrication alteration.

**Fig 13:**
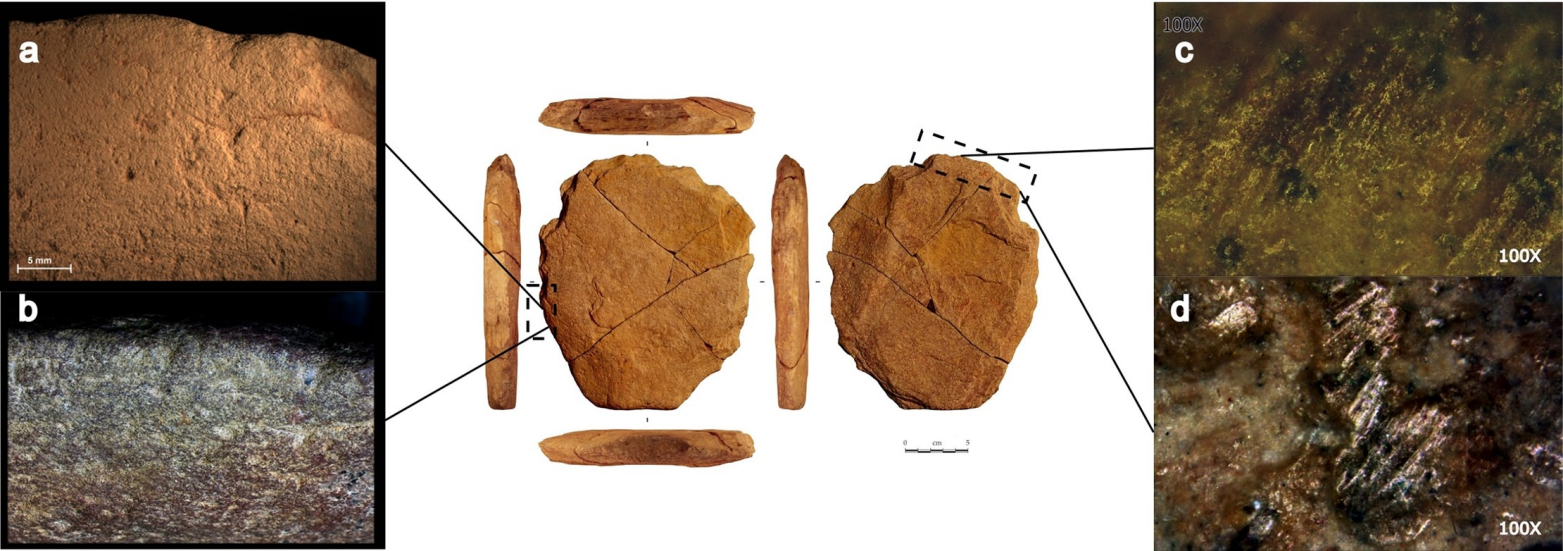
Traceological analysis of the artefact n° 255660.

The **fifth and last stage** corresponds to the production of two new major notches on the quadrangular area from the other surface that break the previous functional balance. They cause a partial exteriorization of the cutting edge called *rostrum* type that is off-centre with respect to the morphological axis of the peduncle area. On this *rostrum* cutting edge, the traceological analysis indicates grooves perpendicular to the edge, as a result of contact with hard material (Fig 13C and 13D). This probably produces a catachresis which no longer seems to take into account the peduncle in the same way as previous stages do.

## Discussion and conclusions

### Vale da Pedra Furada site in the context of early prehistory of South America

The last fifteen years have witnessed a profusion of archaeological and genetic studies that have revolutionized our knowledge of the early occupation of the Americas, without this implying a consistent dialogue between them. In this context, a large part of prehistoric full genome studies converges towards the hypothesis of a peopling of the Americas no greater than 17.5 cal kyr BP. This because at some point between 17.5 and 14.6 cal kyr BP there was a genetic branching between the Ancient Beringians (AB), and Northern Native Americans (NNA)/Southern Native Americans (SNA) populations. Southern Beringia, specifically under the ice sheets that covered present-day Canada, is the proposed place for the NNA-SNA split by the predominant theoretical model [29, 65, 66]. Therefore, the so-called “Beringian Standstill Hypothesis” [67, 68], mainly based in mitochondrial DNA has been reconfigured in time depth (the genetic branching between ancestral Native American populations and their Asian ancestors is hypothesized to be no earlier than 23 kyr BP) [69], but continues proposing a single migration into the Americas occurred between 14 and 16 kyr BP. If this data comes primarily from biology, there is another equally important data register, which is based on the material culture of human societies to attest to their existence. These data attest to human occupation during Marine Isotope Stage 3 (57 and 29 cal kyr BP [70]), specifically during the Dansgaard-Oeschger 16 event when the Bering land bridge, the coastal route and the interior land route were available for a possible passage of human groups, considering the most recent estimates of sea and ice levels [71, 72]. Thus, if there is no genetic data, their absence cannot call into question the cultural material facts. Genetics responds to the question of *who*, material culture responds to *what*, *how* and *why*. What would we do with genetic data alone to talk about humans and humanity?

The main criticism of this alternative model is that there is currently no cultural evidence in western Beringia greater than 32 cal kyr BP, corresponding to that found at the Yana RHS site [73]. It should not be forgotten that these regions during the last glaciation and more particularly during the LGM, are subject to periglacial influences, which will lead to taphonomic processes very often going to the destruction of the sites. As in all other regions of the world, periglacial zones very rarely deliver sites and even fewer intact sites. Human occupations during MIS 3 and 4 of these areas are likely to have been completely destroyed, compared to what is happening in other parts of the world. Therefore, from there to finding LGM or pre-LGM genetic remains will require a lot of luck. However, the fact that they have not been found yet does not mean that they do not exist. Interestingly, a recent multiproxy study has found evidence of last Glacial human presence in the environs of the Lake E5 (Alaska’s North Slope, Eastern Beringia) dated to 32 cal kyr BP, thus supporting the Beringian Standstill Hypothesis [74]. But beyond what we can find and study in the future, it is necessary to take into account that genetic populations do not match archaeologically defined cultures and artefact complexes, and that paleo-demographic interpretations supported by molecular clocks should be taken with extreme caution [75].

Recent investigations in the Northwest of Mexico have reported the finding of cultural evidence dated at least 31 cal kyr BP in the Chiquihuite cave (Zacatecas, Mexico) [76]. In parallel, a statistical study of chronometric data from 42 sites in North America and Beringia concludes that North America was populated before, during and after the LGM [77]. These studies are in addition to what has already been pointed out by previous research [78], which give visibility to a handful of sites in North America dated between 16 and 31 cal kyr BP and which up to now find resistance to be entirely accepted, such as Bluefish cave II (Yukon, Canada), some Buttermilk Creek sites (Texas, USA), some Chesrow complex sites (southeast Wisconsin, USA) Meadowcroft (southwestern Pennsylvania, USA), Miles Point (Maryland, USA), Cactus Hill (Virginia, USA) and Page-Ladson 6 (Florida, USA).

All these evidences are added to the rich and different Pleistocene archaeological record of South America, which includes a multiplicity of cultural evidences dated before, during and immediately after the LGM, coming mainly from sites such as Monte Verde [6, 9, 11], Pilauco [28], Huaca Prieta [7], Pikimachay [79], Taima-Taima [2] and Santa Elina [22]. Likewise, in other regions of South America, such as southern South America, different research teams have been developing important contributions to our understanding of other aspects of the Peopling of the Americas such as timing, settlement patterns, human biogeography, huma-megafauna interactions, etc. [80–82]. Our investigations have revealed the earliest occupations in South America in the Piauí region (Northeast of Brazil), dating at least 40 cal kyr BP. The maximum dating of 41 cal kyr BP corresponds to the archaeological horizons C12 and C13 of the Vale da Pedra Furada site, and is supported by a solid chronostratigraphic sequence (more than 50 dates between 14C and OSL) comprised between 41 and 5 cal kyr BP, in the larger context of the Serra da Capivara region, which so far contains nine sites with pre, pleni and post-LGM cultural strata.

As has been noted elsewhere [13, 14], VPF is an open-air site that presents a stratigraphy of more than 3.30 m containing a succession of archaeological layers separated by sterile sedimentary horizons. Both the typological analysis, production methods and types of raw materials used all differ in sequence. Currently we distinguish at least four major categories of assemblages between 12 and 41,000 years old. Given the presence of boulders in much of the stratigraphic sequence (Fig 2), especially from the C7 layer down, it has been assumed that VPF testifies to a high sediment deposition energy. However, the energy of the ephemeral alluvial streams was not enough to move the coarse material, producing deposits where the last remobilized sediment was sand. As we have said on other occasions, the large boulders are not genetically related to the alluvium, and already represent either the remains of a previous colluvium or human transport. This allows us to point out that the archaeological remains were outside both low-energy colluvial dynamics and ephemeral alluvial events, and therefore, that human occupation occurred on a stable geological substrate. In this context and considering the number of artefacts and the diversity of activities represented by previous traceological analyses, it is very likely that VPF is a place of long human occupation, where sedimentary, climatic and even technical stability are achieved. Probably, the changes between technical facies are correlated to the local climatic instability. The cultural evaluation on a macro-regional scale of these technical changes occupies our current work.

### The exceptionality of the artefact N° 255660

The C7 layer, which occupies the attention of this work, is composed of three sedimentary subsets contained between the C6γ and C8 layers, which do not present cultural remains. C7γ has been dated directly through a ^14^C dating, and indirectly through five OSL dates referring to C7α and C7β and three ^14^C dates referring to C7α. All these dates place C7γ in a period no greater than 27 cal kyr BP and no less than 24 cal kyr BP. The proper dating of levels underlying C7γ confirm this temporal circumscription. In the 6 m^2^ excavation of C7γ-a, 2,200 artefacts were recorded. The techno-functional analysis allowed us to identify techno-types of tools representative of the entire C7 sedimentary complex, particularly bifacial pieces with convergent edges, *rostrums* and *becs* made on quartz and quartzite pebbles. The technical operations used to produce these tools are three: affordance, debitage and shaping. These three operations act in a synergic way in order to establish techno-functional criteria appropriate to the structural configuration of each techno-type. At the same C7γ-a level, artefact N° 255660 represents a technical outlier due to the following particularities: (1) its age; (2) its raw material (silty sandstone) different from the quartz and quartzite pebbles that compose the same level; (3) its incomparable production sequence in the Serra da Capivara region, evidencing a rather complex instrumental biography; and, last but not least, (4) its irrefutable anthropogenic character. The techno-functional characteristics of the VPF silty sandstone plate does not conform to any other artefact observed in Paleoamerican occupations. Five stages of transformations indicate that at least two different objects were established. The first is hexagonal in shape with a double peripheral bevel opposed to a prehensive part, while the second corresponds to a well-known VPF tool called rostrum, representing therefore a catachresis (a deviation from the primary function).

Data reported in all periods and regions of South America do not allow a direct comparison in terms of function and use. Present data shows that VPF silty sandstone plate, securely dated to ~ 24.0 cal kyr BP, that is contemporary to the time of the LGM, is one of the oldest, if not the oldest record of bifacial production in South America. Recent findings in the Chiquihuite cave in Northern Mexico report a single bifacial preform in stratum 1223 on calcareous rock dated 27929 ± 82 14C yr BP [76]. It is clear, then, that the bifacial phenomenon in the Americas has a long history that we still do not fully understand. The term "bifacial" has become a *lingua franca*, everyone uses it, but nobody really knows what it is. If we use the term, we should also begin to be interested in the complete set of artefacts that fall within this denomination and not only the most spectacular. In short, our scientific language does not allow us to address the totality of what we abusively call biface. When examining Holocene industries [82–86], within this same phenomenon and in relation to the artefact n° 255660 it is possible to evoke shovels and hoes, however, these tools are generally made on hard rocks (basalts, andesites and dacites), and in some cases on hard metamorphic or sedimentary rocks. Probably due to this material constraint, the VPF plate also hosted different usage functions. In the context of Piauí lithic industries, the bifacial configuration of this artefact is not an extraordinary aspect. Many tools made of quartz and quartzite are shaped on both surfaces without being called “bifacial pieces”. The pieces that we report here is actually conceived as a bifacial piece. Its singularity lies in the constant search for a double bevel constituting then a transverse techno-functional intention at all transformation stages.

The VPF plate precedes in 6,000 years the records accepted by the current paradigm of post-glacial peopling of South America. Given the current lack of knowledge on the technical and temporal relationship between unifacial and bifacial industries in Pleistocene South America, where pebble and flake industries are largely neglected as simple or expedient technologies, the VPF plate is positioned as a technical outlier, and probably, as the object under whose shadow “simple technologies” will be reconsidered in current models. This artefact confirms the existence of human occupations at 24 cal kyr BP in South America. These populations possess a panoply of objects, which reflect a rich and diversified material culture, like any other human society.

## Supporting information

S1 File (DOCX)
