# Supplementary material for: 24.0 kyr cal BP stone artefact from Vale da Pedra Furada, Piauí, Brazil: Techno-functional analysis
Source: PLoS One. 2021 Mar 10;16(3):e0247965. doi: 10.1371/journal.pone.0247965 (PMC7946292; doi:10.1371/journal.pone.0247965)
Supplement: S1 File — (DOCX) [file pone.0247965.s001.docx]

# S1 File.

| sample identification | | | | raw data | | calibrated data | |
| --- | --- | --- | --- | --- | --- | --- | --- |
| Fumdham ID | layer | lab ID | | conv. 14C age | | calibrated age ± 2 sigma | median proba |
|  |  | chemistry | measurement | age [BP] | ± 1sigma | [cal BP] (proba distribution) | [cal BP] |
| 209523 | C2b | Gif-13102 | SacA37775 | 4790 | 30 | [7536 - 7398] (79.1%) [7333 - 7278] (16.3%) | 5512 |
| 192487 | C2a/b | Gif-12705 | SacA25553 | 6190 | 35 | [7166 - 6936] (94.4%) [6918 - 6904] ( 1.1%) | 7051 |
| 192047 | C2a | Gif-12704 | SacA25552 | 7875 | 40 | [10922 - 10912] ( 0.5%) [10884 - 10870] ( 0.8%) [10809 - 10783] ( 1.9%) [10728 - 10466] (90.2%) [10448 - 10409] ( 2.1%) | 8623 |
| 244358 | C3a/C2 | Gif-13151 | SacA42020 | 11080 | 60 | [13098 - 12836] (94.2%) [12806 - 12792] ( 1.3%) | 12982 |
| 198880 | C3α | Gif-12925 | SacA30854 | 13430 | 50 | [16311 - 15940] (95.4%) | 16125 |
| 191302 | C3β | Gif-12926 | SacA30855 | 13740 | 60 | [16857 - 16350] (95.4%) | 16580 |
| 245182 | C3δ | Gif-13152 | SacA42021 | 14270 | 80 | [17686 - 17646] ( 0.8%) [17528 - 17046] (94.6%) | 17282 |
| 191373 | C4 | Gif-12924 | SacA30853 | 13590 | 60 | [16582 - 16152] (95.4%) | 16356 |
| 257124 | C5 | GifA-18150 | ECHo 2239 | 19500 | 110 | [23774 - 23129] (95.4%) | 23462 |
| 257463 | C5/C6 | GifA-18151 | ECHo 2240 | 20250 | 120 | [24625 - 23905] (95.4%) | 24273 |
| 192452 | C7α | Gif-12837 | SacA28290 | 19970 | 100 | [24201 - 23790] (95.4%) | 23961 |
| 192455 | C7α | Gif-12836 | SacA28289 | 20070 | 100 | [24265 - 23824] (95.4%) | 24039 |
| 192448 | C7α | Gif-12706 | SacA25554 | 20090 | 120 | [24406 - 24396] ( 0.2%) [24375 - 23802] (95.2%) | 24061 |
| 236687 | C7β | Gif-13100 | SacA37773 | 19990 | 110 | [24226 - 23792] (95.4%) | 23981 |
| 232097 | C7γ_α_ | Gif-12923 | SacA30852 | 19700 | 100 | [23870 - 23662] (41.5%) [23642 - 23337] (53.9%) | 23569 |
| 258120 | C7γ_α_ | GifA-18152 | ECHo 2241 | 19180 | 120 | [23710 - 23580] ( 5.6%) [23386 - 22872] (89.9%) | 23077 |
| 237199 | C8 | Gif-13101 | SacA37774 | 29820 | 310 | [35036 - 35004] ( 0.3%) [34920 - 33639] (95.1%) | 34297 |
| 258202 | C8 | GifA-18154 | ECHo 2243 | 29880 | 350 | [35166 - 33692] (95.4%) | 34349 |

**S1 Table. ^14^C data from Vale da Pedra Furada site.** Calibration was performed based on SHCal20 (Hogg et al. 2020) curve, using ChronoModel software (Lanos and Dufresne, 2019).

| MG-OSL | | | CAM | | | | | |
| --- | --- | --- | --- | --- | --- | --- | --- | --- |
| Sample | Location | n aliquots (passed all tests) | D_e_ (Gy) | 𝞂 | OD (%) | 𝞂 | Age (ka) | 𝞂 |
| BR2011-08 | C2a | 28 | 8.5 | 0.2 | 10 | 2 | 7.5 | 0.8 |
| BR2011-09 | C2b | 47 | 10.8 | 0.2 | 11 | 1 | 9.1 | 1 |
| BR2015-01 | C3α | 28 | 10.7 | 0.1 | 4 | 0.8 | 15.9 | 2.2 |
| BR2011-11 | C3δ/ε | 42 | 16.0 | 0.2 | 8 | 1 | 19.5 | 2 |
| BR2015-02 | C3ν | 30 | 15.03 | 0.12 | 3 | 0.8 | 23.1 | 2.1 |
| BR2011-12 | C4 | 40 | 18.0 | 0.2 | 5 | 1 | 18.7 | 1.7 |
| BR2015-03 | C4 | 30 | 17.85 | 0.13 | 2.6 | 0.8 | 17.7 | 2.2 |
| BR2015-04 | C5 | 26 | 19.31 | 0.18 | 3.5 | 0.9 | 20.4 | 2.5 |
| BR2011-13 | C5 | 29 | 18.6 | 0.2 | 5 | 1 | 22.6 | 2.4 |
| BR2015-05 | C6top | 27 | 22.78 | 0.17 | 1.9 | 1.1 | 23.8 | 2.9 |
| BR2011-14 | C7α | 29 | 21.4 | 0.3 | 7 | 1 | 27.6 | 2.8 |
| BR2011-15 | C7α | 46 | 21.2 | 0.3 | 7 | 1 | 23.7 | 2.6 |
| BR2015-06 | C6base | 31 | 21.34 | 0.12 | 0 | 0.0 | 22.5 | 2 |
| BR2011-32 | C7α | 36 | 24.0 | 0.3 | 3 | 1 | 23.4 | 2.8 |
| BR2012-13 | C7γ | 23 | 22.6 | 0.4 | 6 | 1 | 26.5 | 2.8 |
| BR2012-14 | C10’ | 40 | 53.7 | 0.5 | 4 | 1 | 38.4 | 3.6 |

**S2 Table.**  **OSL data from Vale da Pedra Furada site.** Equivalent dose (D_e_) from multi-grain measurements; number of accepted multi-grain aliquots (passing recycling and recuperation tests) are indicated. OD is the overdispersion, applying the Central Age Model. Ages are given in ka. « C10’ »: it is certainly the C10 layer, but there is no stratigraphic contact with the other C10 excavated in a different sector. In blue: unpublished data.

| SG-OSL |  |  | **CAM** | | | | | | **BaSar** | | | |
| --- | --- | --- | --- | --- | --- | --- | --- | --- | --- | --- | --- | --- |
| Sample | Location | n grains (passed all tests) | De (Gy) | 𝞂 | OD | 𝞂 | Age (ka) | 𝞂 | De (Gy) | 𝞂 | Age (ka) | 𝞂 |
| BR2011-08 | C2a | 99 | 8.1 | 0.5 | 60 | 5 | 5.7 | 0.6 | 7.7 | 0.9 | 6.00 | 0.90 |
| BR2011-11 | C3δ/ε | 62 | 14.2 | 1.2 | 62 | 6 | 14.2 | 1.6 | 14.5 | 1.2 | 13.90 | 1.60 |
| BR2011-15 | C7α | 68 | 21.7 | 1.1 | 33 | 4 | 20.8 | 2.4 | 21.5 | 1.7 | 21.00 | 1.90 |
| BR2011-32 | C7α | 52 | 24.5 | 1.3 | 30 | 4 | 24.7 | 2 | 26.7 | 0.9 | 23.00 | 1.90 |
| BR2013-04 | C8 | 79 | 23.77 | 1.37 | 45 | 4.0 | 34.1 | 2.7 |  |  |  |  |
| BR2013-06 | C11/12 | 25 | 38 | 2.07 | 16 | 6.0 | 41.2 | 5.9 |  |  |  |  |

**S3 Table.** Equivalent dose (D_e_) from single-grain measurements; number of accepted grains (after rejection of poorly suited grains) are indicated. OD is the overdispersion, applying the Central Age Model. Ages are presented in ka. Results are given for both Central Age Model (Galbraith *et al.*, 1999) and BaSAR (Combès *et al.*, 2015). In blue: unpublished data.

|  | Quartz grains | | | | | | sediments | | | | | |
| --- | --- | --- | --- | --- | --- | --- | --- | --- | --- | --- | --- | --- |
| Sample | K (%) | 𝞂 | U (ppm) | 𝞂 | Th (ppm) | 𝞂 | K (%) | 𝞂 | U (ppm) | 𝞂 | Th (ppm) | 𝞂 |
| BR2011-08 | 0.32 | 0.02 | 2.08 | 0.02 | 12.12 | 0.12 | 0.08 | 0.01 | 0.85 | 0.03 | 6.37 | 0.10 |
| BR2011-09 | 0.36 | 0.02 | 2.00 | 0.06 | 12.94 | 0.06 | 0.08 | 0.01 | 0.96 | 0.02 | 7.05 | 0.07 |
| BR2015-01 | 0.01 | 0.00 | 0.04 | 0.00 | 1.53 | 0.15 | 0.05 | 0.00 | 0.67 | 0.01 | 5.27 | 0.05 |
| BR2011-11 | 0.29 | 0.02 | 2.22 | 0.02 | 6.46 | 0.16 | 0.03 | 0.01 | 0.31 | 0.02 | 2.39 | 0.05 |
| BR2015-02 | 0.01 | 0.00 | 0.04 | 0.00 | 1.89 | 0.09 | 0.05 | 0.00 | 0.61 | 0.01 | 4.86 | 0.04 |
| BR2011-12 | 0.01 | 0.00 | 1.80 | 0.01 | 4.09 | 0.08 | 0.07 | 0.01 | 0.78 | 0.02 | 5.44 | 0.08 |
| BR2015-03 | 0.01* | 0.001* | 0.03* | 0.003* | 2.268* | 0.2268* | 0.09 | 0.05 | 1.03 | 0.01 | 7.43 | 0.06 |
| BR2015-04 | 0.01* | 0.001* | 0.03* | 0.003* | 2.268* | 0.2268* | 0.09 | 0.01 | 0.93 | 0.01 | 7.25 | 0.06 |
| BR2011-13 | 0.01 | 0.00 | 2.53 | 0.05 | 5.68 | 0.24 | 0.03 | 0.01 | 0.32 | 0.01 | 2.32 | 0.05 |
| BR2015-05 | 0.01 | 0.00 | 0.04 | 0.00 | 1.98 | 0.20 | 0.086' | 0.005' | 0.933' | 0.012' | 7.246' | 0.06' |
| BR2011-14 | 0.02 | 0.00 | 1.96 | 0.06 | 4.37 | 0.12 | 0.05 | 0.01 | 0.59 | 0.02 | 4.11 | 0.06 |
| BR2011-15 | 0.03 | 0.00 | 1.97 | 0.10 | 8.32 | 0.46 | 0.06 | 0.01 | 0.76 | 0.02 | 5.26 | 0.06 |
| BR2015-06 | 0.01 | 0.00 | 0.05 | 0.00 | 2.40 | 0.24 | 0.08 | 0.01 | 0.97 | 0.01 | 7.37 | 0.06 |
| BR2011-32 | 0.40 | 0.02 | 2.42 | 0.08 | 10.43 | 0.18 | 0.08 | 0.01 | 0.95 | 0.02 | 6.21 | 0.08 |
| BR2012-13 | 0.01 | 0.00 | 2.15 | 0.05 | 4.39 | 0.17 | 0.06 | 0.01 | 0.71 | 0.02 | 5.61 | 0.09 |
| BR2013-04 | 0.00 | 0.00 | 0.01 | 0.00 | 0.02 | 0.00 | 0.10 | 0.01 | 1.18 | 0.03 | 7.97 | 0.10 |
| BR2012-14 | 0.02 | 0.00 | 2.49 | 0.08 | 6.38 | 0.27 | 0.09 | 0.01 | 1.09 | 0.02 | 9.61 | 0.09 |
| BR2013-06 | 0.00 | 0.00 | 0.01 | 0.00 | 5.61 | 0.56 | 0.09 | 0.01 | 1.24 | 0.03 | 10.82 | 0.13 |

**S4 Table. Radiometric data**. K, U and Th of the sediments measured thanks to High Purity Low Background Ge gamma spectrometry, and internal values (K, U and Th) of the quartz grains from ICP-MS measurements (see Lahaye *et al.*, 2015 for more details).

| Phase | Posterior distribution Begin MAP (ka cal BP) (@80%) | Posterior distribution End MAP (ka cal BP) (@80%) | Phase Time Range (80%)  (Age cal BP) |
| --- | --- | --- | --- |
| C2a | 6.9 | 6.1 | 7,279 – 3,933 |
| C2a/b | 7.1 | 7.1 | 7,298 – 6,816 |
| C2b | 9.4 | 7.2 | 10,979 – 6,955 |
| C2-C3 contact | 12.9 | 12.9 | 13,066 – 12,728 |
| C3 | 17.5 | 16.0 | 18,719 – 13,350 |
| C4 | 19.7 | 17.9 | 20,928 – 17,298 |
| C5 | 23.2 | 21.0 | 23,634 – 19,975 |
| C5-C6 contact | 23.4 | 23.4 | 23,829 – 22,886 |
| C6 | 23.7 | 23.6 | 24,021 – 23,086 |
| C7α | 24.2 | 23.8 | 24,679 – 23,305 |
| C7β | 24.4 | 24.3 | 25,271 – 23,971 |
| C7γ | 25.0 | 24.5 | 27,615 – 24,071 |
| C8 | 34.1 | 33.7 | 37,483 – 29,365 |
| C10’ | 38.2 | 38.2 | 42,865 – 34,688 |
| C11-12 | 44.5 | 44.5 | 54,614 – 38,114 |

**S5 Table. Phases time range as from the chronological model.** The starting and ending points of the posterior distribution are defined as the MAP (*Maximum A Posteriori*) of the phase distribution, in each case (second and third columns), at 80% confidence level. The phase time range (fourth column) is also given for a 80 % confidence level.


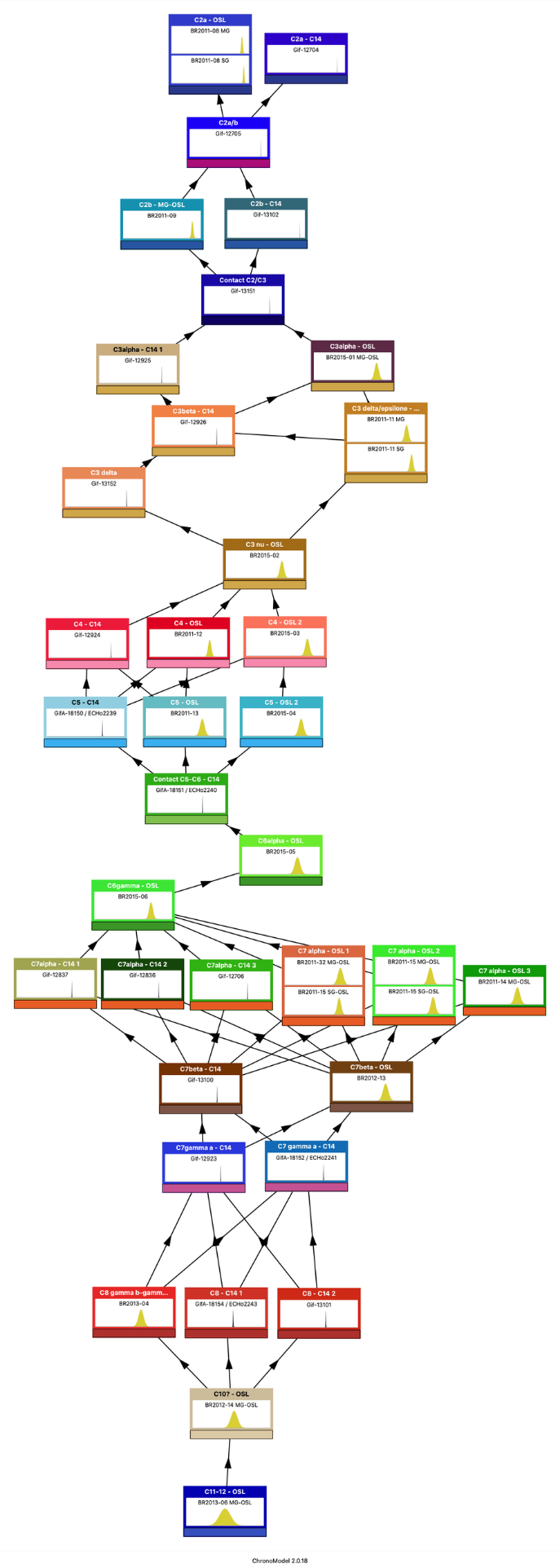

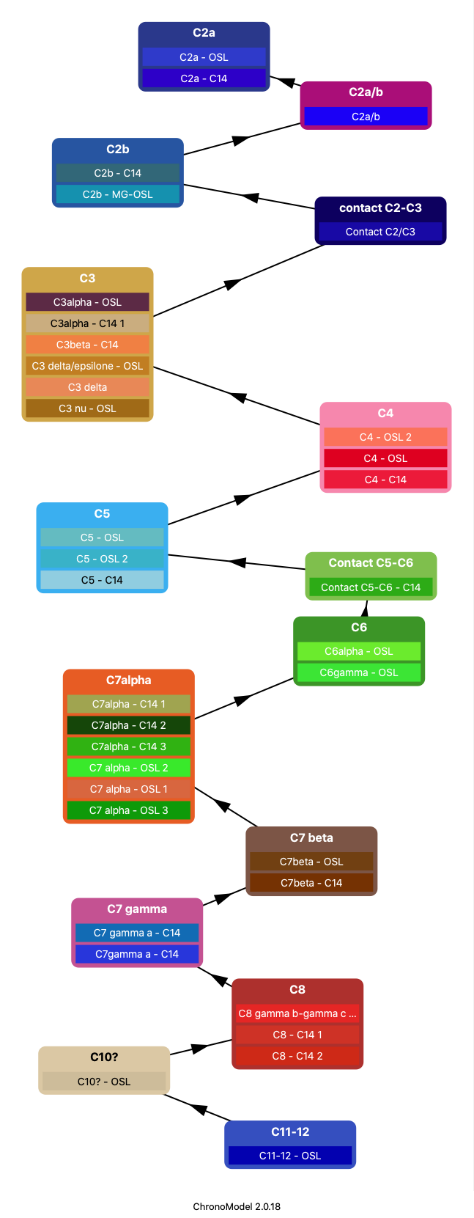


**S1 Fig. Proposed model, events (a) and phases (b) defined**. ChronoModel 2.0.18 software (Lanos and Dufresne, 2019) has been used. Each phase (e.g. C7α) is represented by a colored block within which OSL and ^14^C dating representing it are noted. The chronological links between the phases (stratigraphic succession) are represented by arrows.


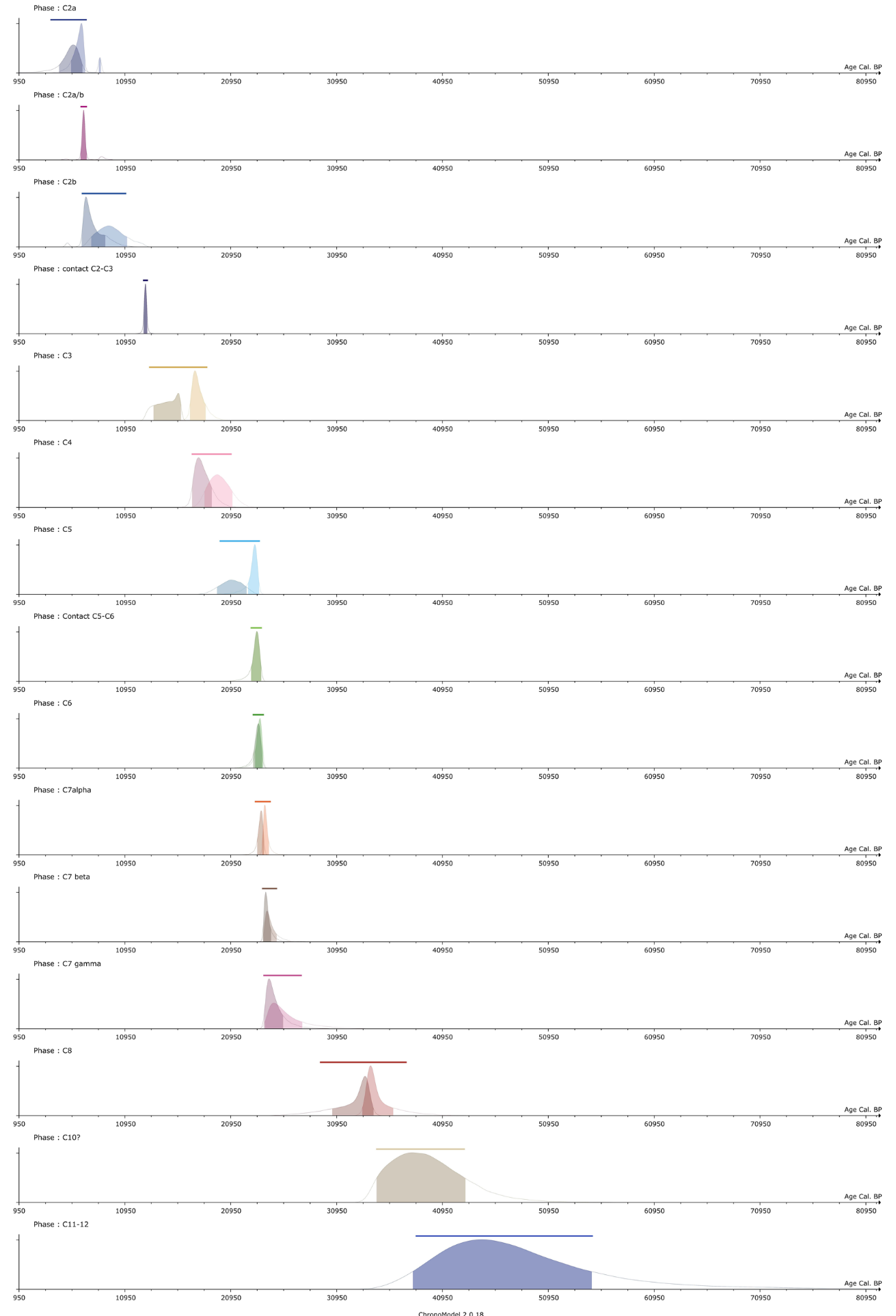


**S2 Fig.** **Posterior distributions of the fifteen defined phases.** Confidence bar are given with a 80% confidence level. Hierarchical Bayesian modeling realized thanks to ChronoModel 2.0.18 software package (Lanos and Dufresne, 2019). When a phase is represented by at least two dating, the posterior probability of the beginning and end of the phase are indicated with probability distributions, the phase duration is symbolized by the bar.

**S3 Fig. 3D model of the VPF shaped silty sandstone plate (artefact N °255660).** Available on the following link https://visual.ariadne-infrastructure.eu/3d/vale-da-pedra-furada-mercurio-text?standalone


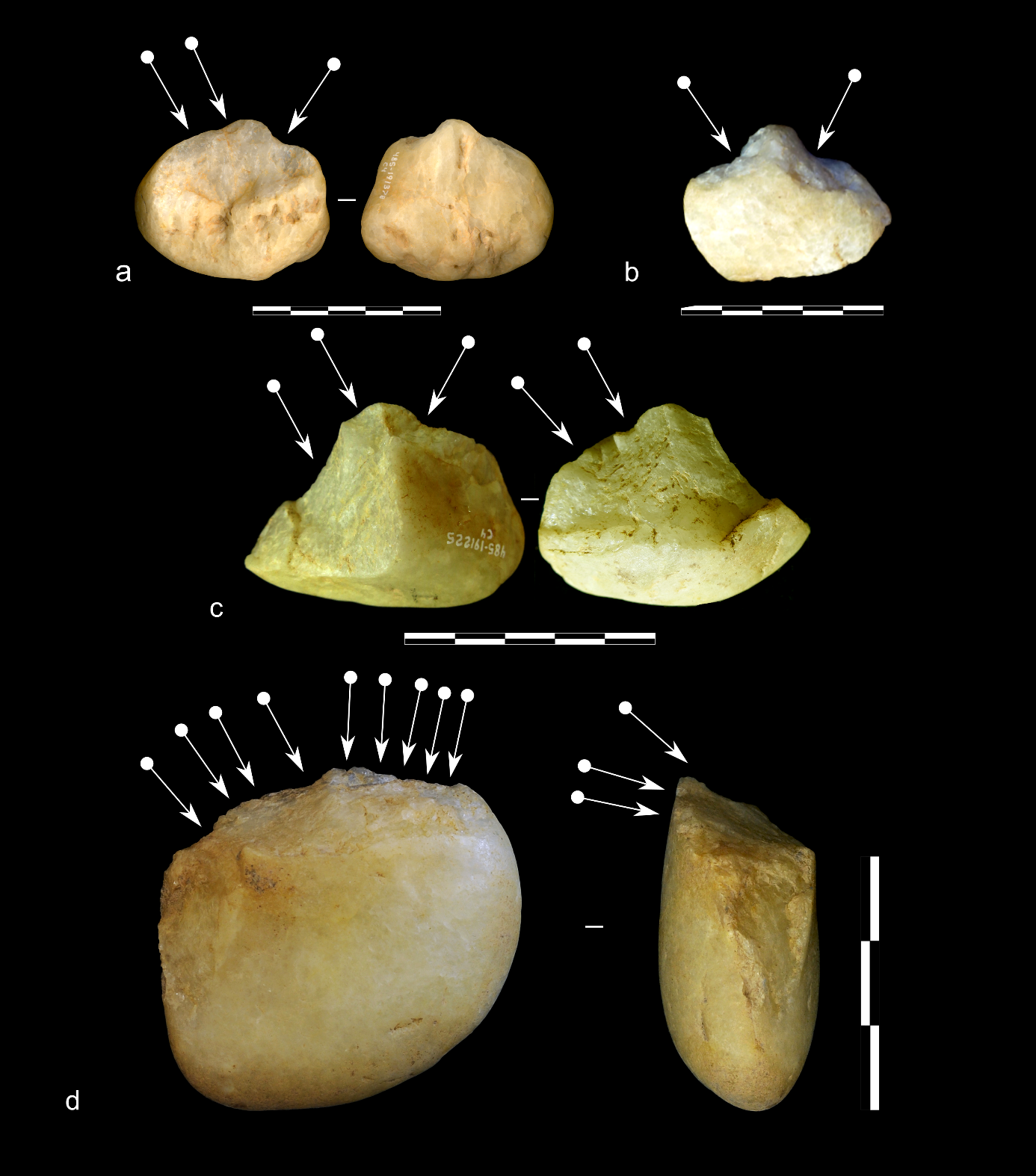


**S4 Fig.**  *Rostrum*-type family: (a) *rostrum*-type cutting-edge on quartz pebble from C4 layer, (b) *rostrum*-type cutting-edge on quartz pebble from C5 layer, (c) *rostrum*-type cutting-edge on quartz pebble flake from C4 layer, (d) *rostrum*-type cutting-edge on quartz pebble from C2a layer. Arrows indicate negatives of removals. Graphic scale in cm.


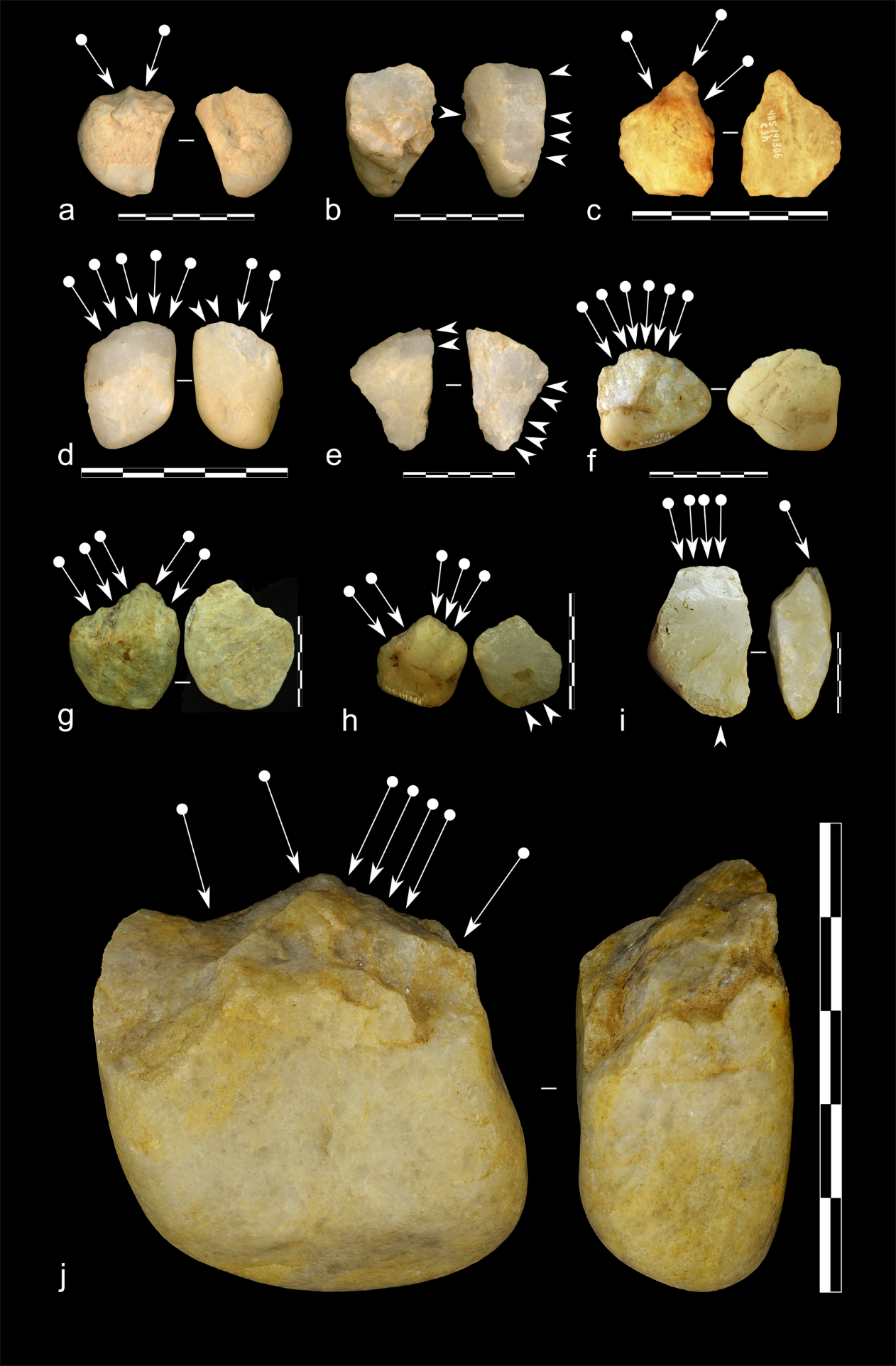


**S5 Fig.** (a) *bec*-type cutting-edge on quartz pebble from C3 epsilon layer, (b) bifacial piece on quartz pebble from C3 epsilon layer, (c) *bec*-type cutting-edge on quartz pebble from C3 eta layer, (d) double bevelled cutting-edge on quartz pebble from C3 epsilon layer, (e) edge-trimmed quartz flake from C3 epsilon layer, (f) *rostrum*-type cutting-edge on quartz pebble from C3 eta layer, (g) unifacial asymmetric convergent cutting-edge on quartz pebble split flake from C3 eta layer, (h) bifacial symmetrical convergent cutting-edge on quartz pebble split flake from C3 delta layer, (i) double bevelled transverse cutting-edge on quartz cobble split flake from C3gamma layer, (j) *rostrum*-type cutting-edge on quartz pebble from C3alpha layer. Arrows indicate negatives of removals. Graphic scale in cm.


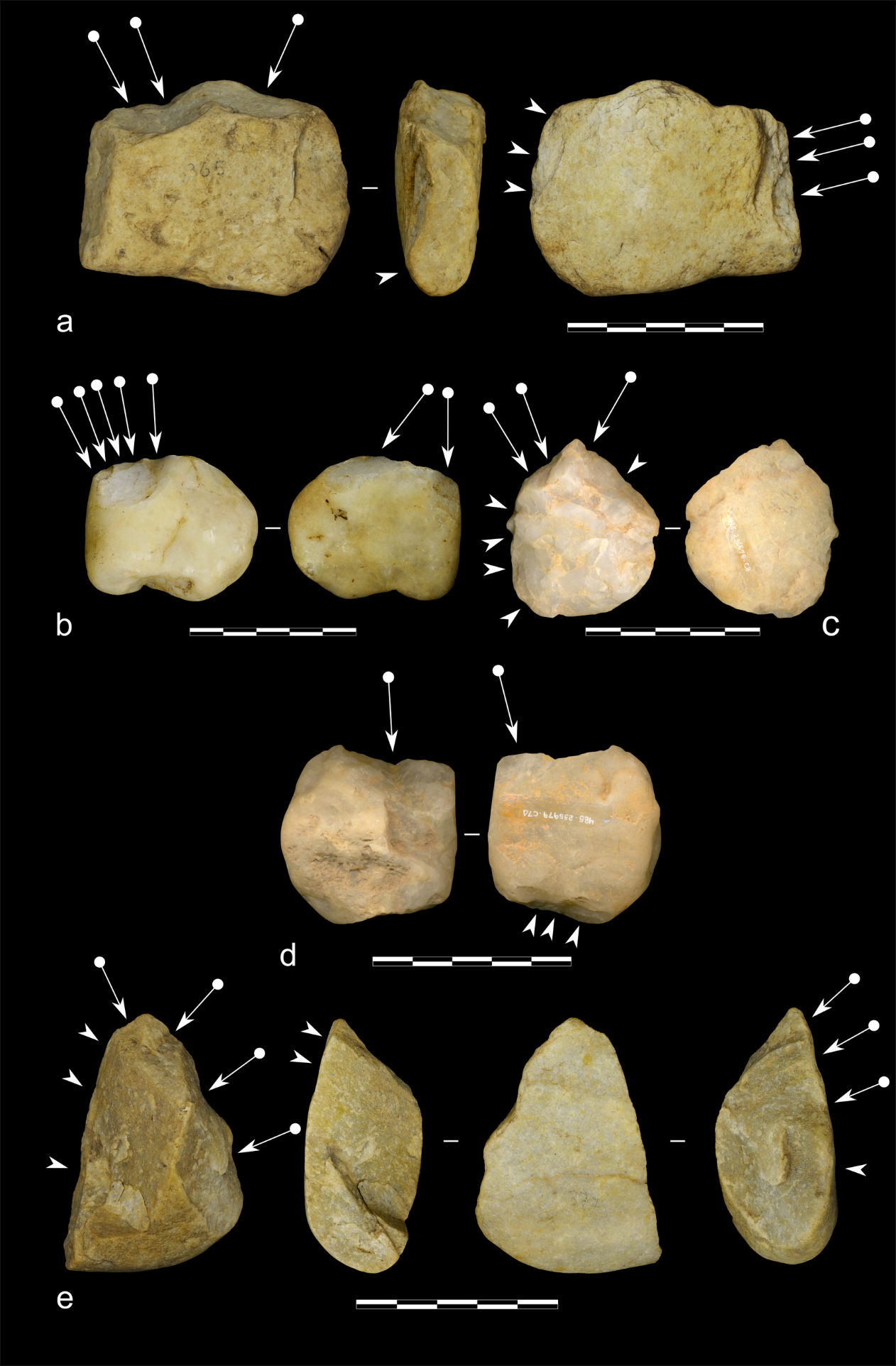


**S6 Fig.** (a) *rostrum*-type cutting-edge on quartz block from C7alpha layer, (b) simple bevelled cutting-edge on quartz pebble from C7alpha layer, (c) *bec*-type cutting-edge on quartz pebble flake from C7/C8 contact layer, (d) simple bevelled cutting-edge on quartz pebble from C7delta layer, (e) unifacial asymmetric convergent cutting-edge on quartzite split cobble from C7alpha layer. Arrows indicate negatives of removals. Graphic scale in cm.


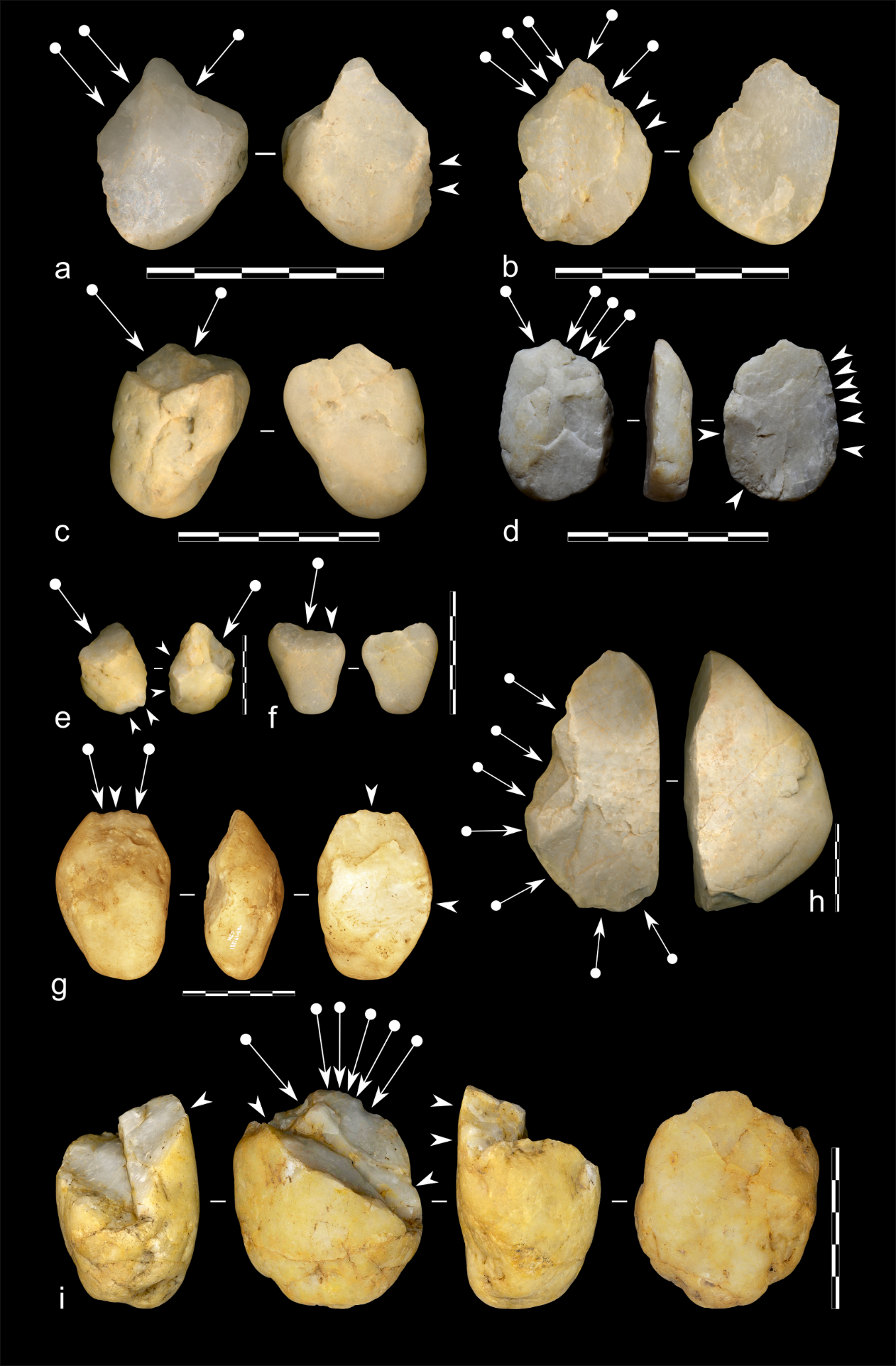


**S7 Fig.** (a) *Rostrum*-type cutting-edge on quartz pebble from C7alpha layer, (b) *bec*-type cutting-edge on quartz pebble flake from C7alpha layer, (c) *bec*-type cutting-edge on quartz pebble from C7alpha layer, (d) inverse retouch and *bec* on split from C7alpha layer, (e) bifacial piece on quartz pebble from C7gamma-a, (f) simple bevelled cutting-edge on quartz pebble from C7alpha layer, (g) double levelled transversal cutting-edge on quartz pebble from C7alpha layer, (h) denticulate cutting-edge on quartzite cobble from C7alpha layer, (i) rostrum-type cutting-edge on quartz pebble from C7alpha layer. Arrows indicate negatives of removals. Graphic scale in cm.


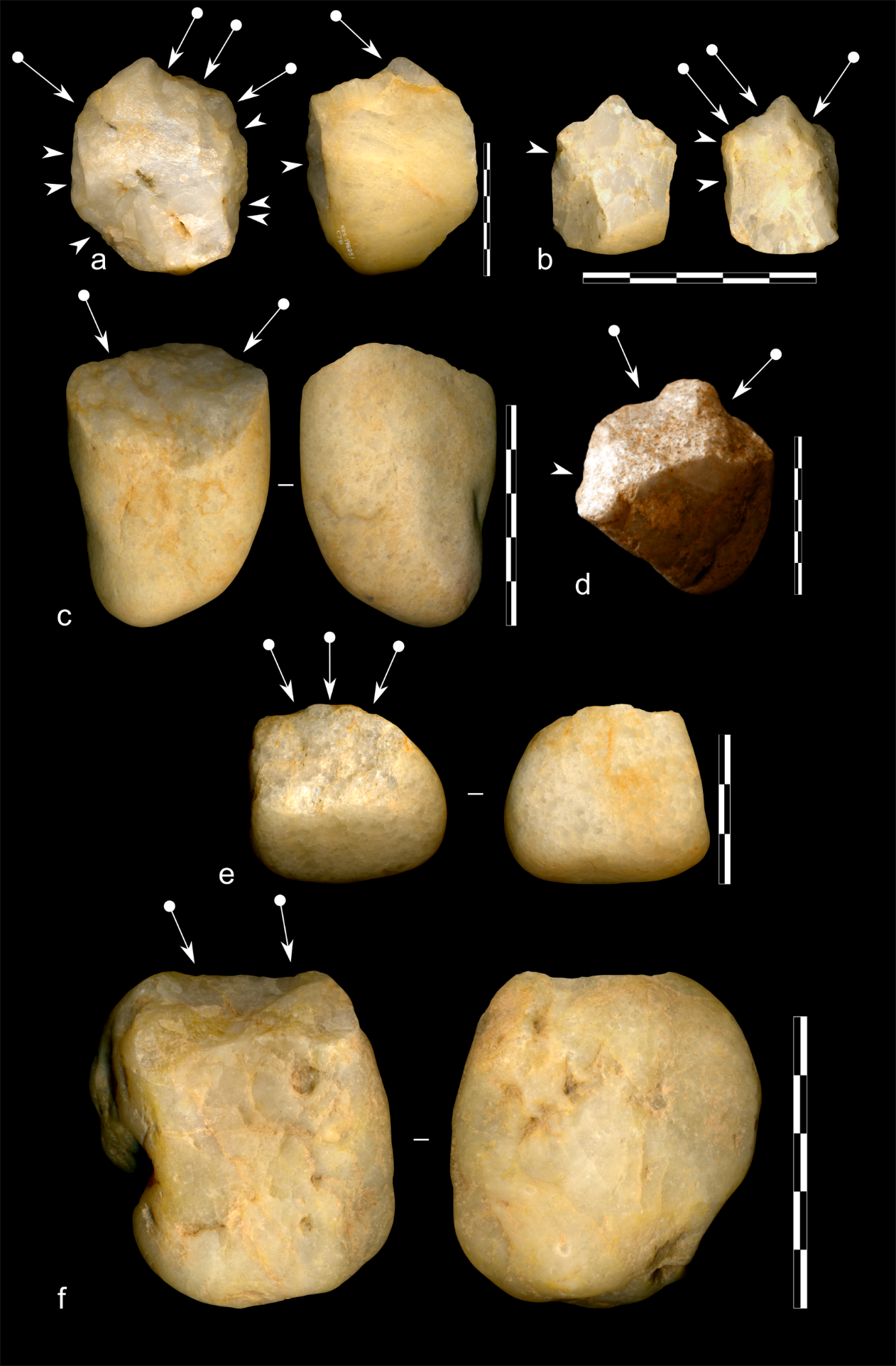


**S8 Fig.** (a) *bec*-type cutting-edge on quartz cobble from C7beta layer, (b) *bec*-type cutting-edge on quartz pebble from C7beta layer, (c) simple bevelled cutting-edge on quartz cobble from C7beta layer, (d) *rostrum*-type cutting edge on quartz pebble from C7beta layer, (e) *rostrum*-type cutting-edge on quartz pebble from C7beta layer, (f) simple bevelled cutting-edge on quartz pebble from C7beta layer. Arrows indicate negatives of removals. Graphic scale in cm.


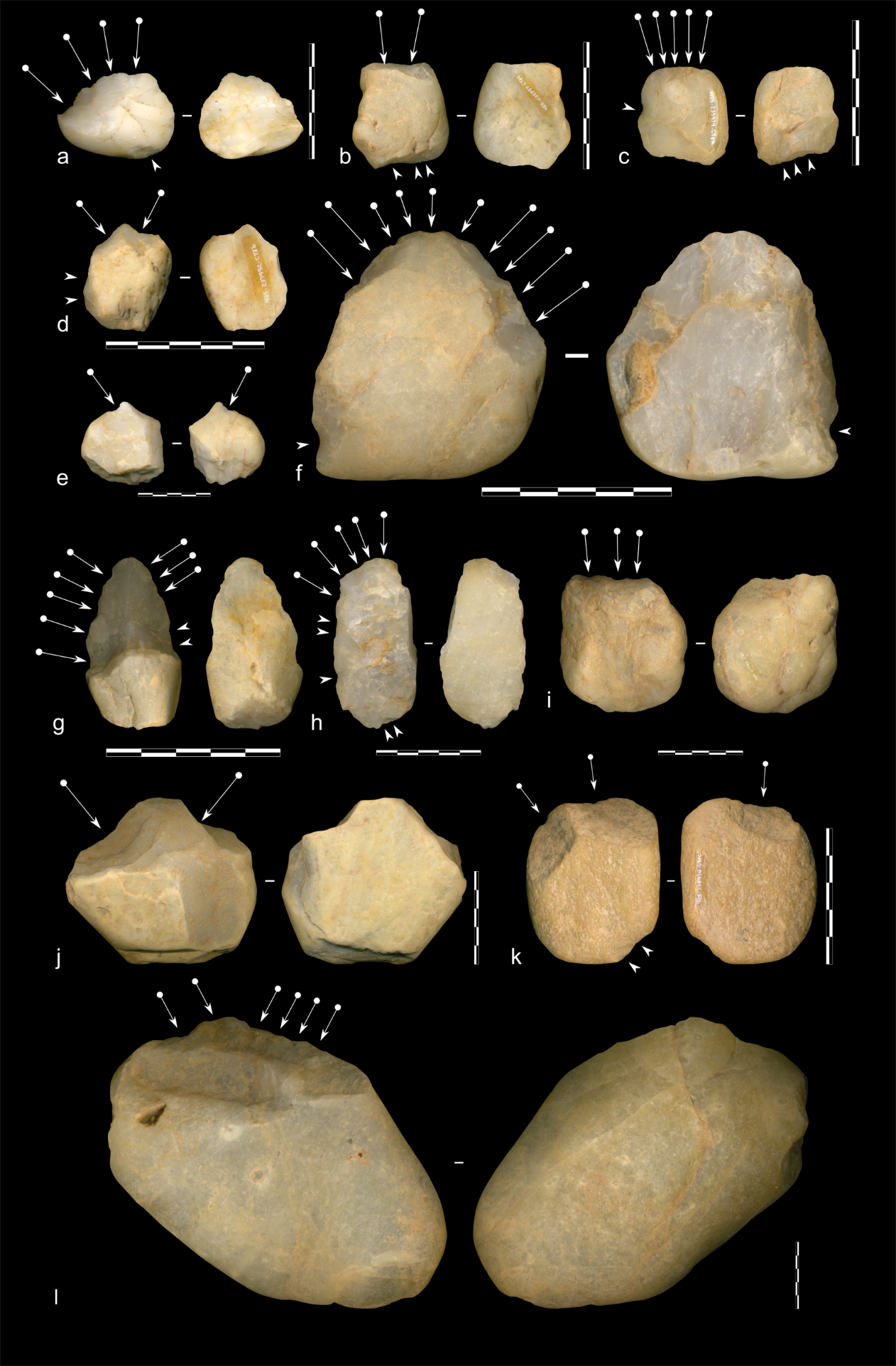


**S9 Fig.** (a) denticulate cutting-edge on quartz pebble flake from C7gamma layer, (b) simple bevelled cutting-edge on quartz pebble from C7gamma-c layer, (c) simple bevelled cutting-edge on quartz pebble flake from C7gamma-a layer, (d) *bec*-type cutting-edge on quartz pebble from C7gamma-b layer, (e) *bec*-type cutting-edge on quartz pebble from C7gamma-c layer, (f) unifacial piece on quartz cobble flake from C7gamma layer; (g) unifacial piece on quartz pebble from C7gamma layer, (h) unifacial piece on quartz pebble flake from C7gamma layer, (i) denticulate cutting-edge on quartz cobble from C7gamma-c layer; (j) *rostrum*-type cutting-edge on quartzite cobble from C7gamma layer; (k) bifacial piece on quartzite pebble from C7gamma-c layer; (l) *rostrum*-type cutting-edge on quartz cobble from C7gamma-a layer. Arrows indicate negatives of removals. Graphic scale in cm.


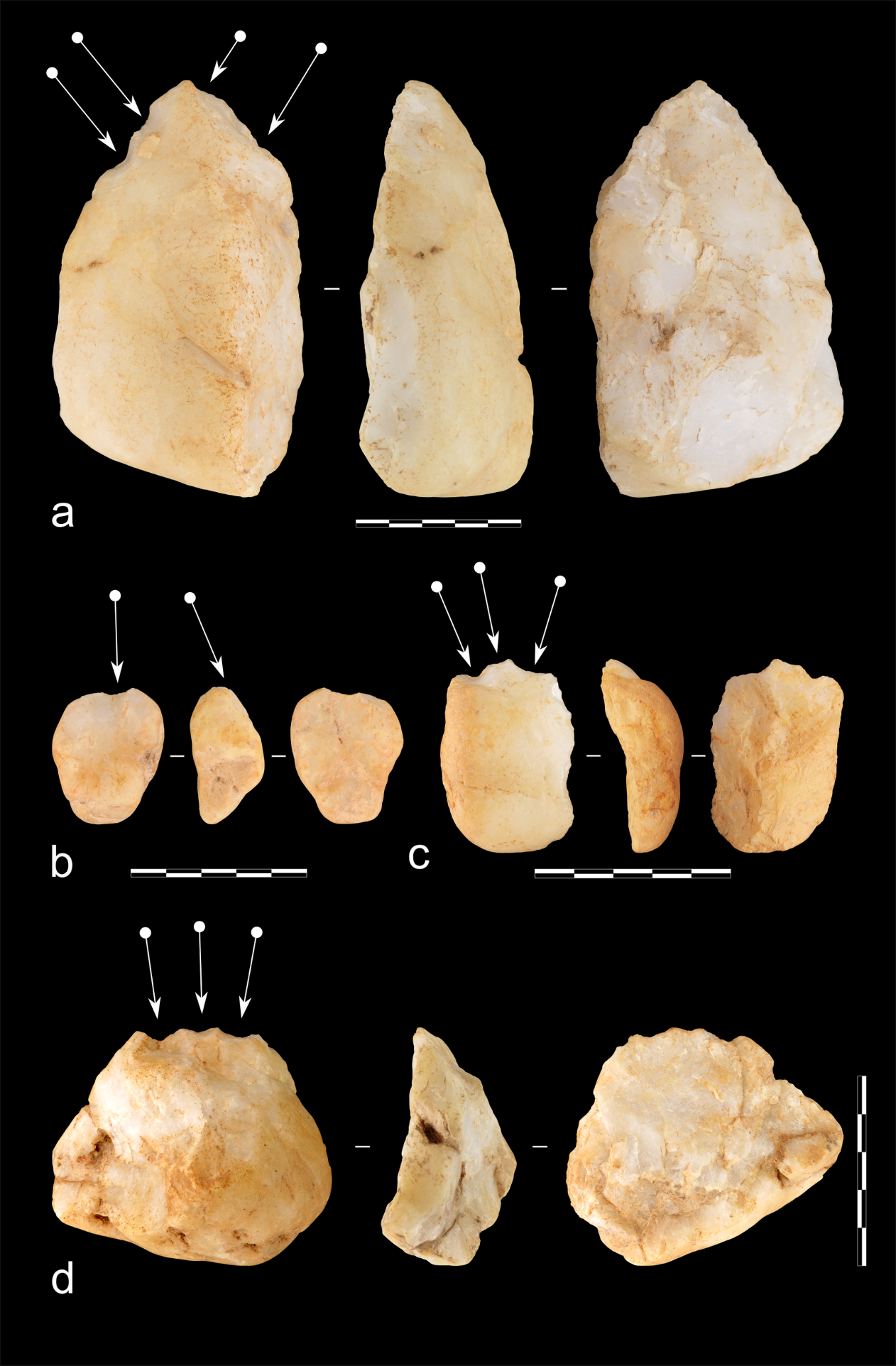


**S10 Fig.** (a) symmetrical convergent cutting-edge on quartz cobble flake from C11alpha layer, (b) simple bevelled cutting-edge on quartz pebble from C11alpha layer, (c) *bec*-type cutting-edge on quartz pebble flake from C13 layer, (d) denticulate transversal cutting-edge on quartz pebble flake from C12 layer. Arrows indicate negatives of removals. Graphic scale in cm.

# References

Combès B, Philippe A, Lanos P, Mercier N, Tribolo C, Guerin G, et al. A Bayesian central equivalent dose model for optically stimulated luminescence dating. Quaternary Geochronology. 2015;28: 62–70. doi:10.1016/j.quageo.2015.04.001

Galbraith RF, Roberts RG, Laslett GM, Yoshida H, Olley JM. Optical Dating of Single and Multiple Grains of Quartz from Jinmium Rock Shelter, Northern Australia: Part I, Experimental Design and Statistical Models*. Archaeometry. 1999;41: 339–364. doi:10.1111/j.1475-4754.1999.tb00987.x

Hogg A.G., Heaton T.J., Hua Q., Palmer J.G., Turney C.S.M., Southon J., Bayliss A., Blackwell P.G., Boswijk G., Bronk Ramsey C., Pearson C., Petchey F., Reimer P., Reimer R., Wacker L. (2020) SHCal20 Southern hemisphere calibration, 0-55,000 years cal BP. Radiocarbon 10.1017/rdc.2020.59, 1-20.

Lanos P., Dufresne P. (2019) ChronoModel version 2.0: Software for Chronological Modelling of Archaeological Data using Bayesian Statistics, 2.0 ed.
